# Total Triterpenes of *Wolfiporia cocos* (Schwein.) Ryvarden & Gilb Exerts Antidepressant-Like Effects in a Chronic Unpredictable Mild Stress Rat Model and Regulates the Levels of Neurotransmitters, HPA Axis and NLRP3 Pathway

**DOI:** 10.3389/fphar.2022.793525

**Published:** 2022-02-14

**Authors:** Xiang Pan, Kezhuo Chen, Sijie Han, Xinyao Luo, Dandan Zhang, Hanrui Zhang, Lian Zhang, Xuxiang Zhou, Jing Li, Jingxian Fang, Shiqin Wang, Xiaochuan Ye

**Affiliations:** Hubei Key Laboratory of Resources and Chemistry of Chinese Medicine, School of Pharmacy, Hubei University of Chinese Medicine, Wuhan, China

**Keywords:** depression, triterpenes, chronic unpredictable mild stress, neurotransmitter, hypothalamic-pituitary-adrenal axis, NLRP3 pathway, Wolfiporia cocos (schwein.) ryvarden & gilb

## Abstract

**Purpose:** *Wolfiporia cocos* is frequently used in traditional Chinese medicine to treat depression. However, antidepressant-like effects of the main active ingredients of *Wolfiporia cocos*, total triterpenes of *Wolfiporia cocos* (TTWC), are not well studied. This study aimed to investigate those effects and explore their specific mechanisms of action in depth.

**Methods:** Chemical components of TTWC were analyzed using LC-MS. Depression-like behavior in rats were induced by chronic unpredictable mild stress (CUMS). The suppressive effects of TTWC (60, 120, 240 mg/kg) against CUMS-induced depression-like behavior were evaluated using the forced swimming test (FST), open field test (OFT) and sucrose preference test (SPT). Levels of 5-hydroxytryptamine (5-HT), glutamate (GLU), corticotropin-releasing hormone (CRH), interleukin-1 beta (IL-1beta), interleukin-18 (IL-18), interleukin-6 (IL-6), and tumor necrosis factor-alpha (TNF-alpha) in different groups were determined by ELISA. Western blotting (WB) was used to detect the expression of NLRP3, ASC, pro-caspase-1, caspase-1, pro-IL-1beta, IL-1beta, pro-IL-18, and IL-18 in the prefrontal cortex. Additionally, the mRNA levels of NLRP3, ASC, caspase-1, IL-1beta and IL-18 were detected by RT-PCR.

**Results:** A total of 69 lanostane-type triterpene acids of TTWC were identified. The results showed that TTWC exhibited an antidepressant-like effect in CUMS rats, reversed the decreased sugar preference in the SPT, reduction of immobility time in the FST, reduced the rest time, increased the total moving distance in the OFT. TTWC increased 5-HT levels and decreased GLU levels in the hippocampus. Moreover, TTWC decreased CRH levels in serum, indicating the regulation of over-activation of the hypothalamic-pituitary-adrenal (HPA) axis. In addition, reduced serum levels of IL-1beta, IL-18, IL-6, and TNF-alpha. The WB results implied that TTWC inhibited the expression of NLRP3, ASC, caspase-1, IL-1beta, and IL-18 in the prefrontal cortex and enhanced the expression of pro-caspase-1, pro-IL-1beta, and pro-IL-18. Although most of the results were not significant, PCR results showed that TTWC inhibited the expression of NLRP3, ASC, caspase-1, IL-1beta, and IL-18 in the prefrontal cortex.

**Conclusion:** TTWC treatment exerted an antidepressant-like effect and regulates neurotransmitters, HPA axis and NLRP3 signaling pathway. These results indicated the potential of TTWC in preventing the development of depression.

## Introduction

Depression, which is characterized by depressive mood or loss of anhedonia, is one of the most common and costly psychiatric disorders. The global prevalence of depression is high; over 264 million people of all ages worldwide suffer from depression, leading approximately 80 thousand patients to commit suicide (www.who.int). Rising competition in modern society has led to a sharp surge in the likelihood of individuals to suffer from depression in recent years (Zhang et al., 2021). Current treatments for depression mainly focus on the monoaminergic system, and the commonly used antidepressants are selective serotonin reuptake inhibitors, or norepinephrine reuptake inhibitors. Unfortunately, studies have shown that one-third of patients do not respond to the initial treatment, and almost half of them have a secondary response, often producing adverse reactions such as sexual dysfunction, nausea, tremor, and insomnia (Li et al., 2020a; Zhuo et al., 2020). To combat depression, the identification of natural medicines with better curative effects and increased safety is urgently needed.

*Wolfiporia cocos* (Schwein.) Ryvarden & Gilb is the dried sclerotium of the polyporaceae fungus *Wolfiporia cocos*, which often grows as a parasite on pine roots (Li et al., 2019). *Wolfiporia cocos,* as a medicinal material and food, has existed for thousands of years in Chinese culture. At present, the secondary response of many antidepressants is inevitable, which on the other hand aggravates the disease of patients. *Wolfiporia cocos*, has the advantages of high safety and large yield, and its development as a therapeutic alternative may reduce the economic burden and physical torture of patients. In modern pharmacological research, it is widely used as a constituent of many formulae (including ding-zhi-xiao-wan, xiao-yao-san), wherein it plays a significant role in the treatment of neurodegenerative diseases (He et al., 2020; Liu et al., 2021). The main active components of *Wolfiporia cocos* are triterpenoids and polysaccharides (including water-soluble polysaccharides and alkali-soluble polysaccharides). Water-soluble polysaccharides exhibit a potent antidepressant-like effect by regulating monoaminergic neurotransmission and inactivation of inflammation (Huang et al., 2020). Similarly, alkali-soluble polysaccharides have exhibited their antidepressant-like effects by regulating neurotransmitters and alleviating neuroinflammation (Chen et al., 2021). Indeed, petroleum ether extract of *Wolfiporia cocos* has been confirmed to improve depression-like behavior by acting on gut microbiota and metabolites in chronic unpredictable mild stress (CUMS) rats (Gao et al., 2020). However, to date, most of the reported triterpenes of *Wolfiporia cocos* have been triterpene acids. Our previous study found only a tiny amount of *Wolfiporia cocos* triterpenes in petroleum ether extraction. Therefore, the possibility of the total triterpenes of *Wolfiporia cocos* (TTWC) exerting antidepressant-like effects is still unknown, and hence worth investigating.

Accumulating evidence shows that brain inflammation may be a critical factor in developing depression (Hodes et al., 2015). The NLRP3 inflammasome pathway, a key target in depression, mediates the production of proinflammatory cytokines such as interleukin-1beta (IL-1beta) and interleukin-18 (IL-18) (Xu et al., 2016). Indeed, proinflammatory cytokines, including IL-1beta, interleukin-6 (IL-6), and tumor necrosis factor-alpha (TNF-alpha), have been implicated in the etiology of depression, contributing to cellular damage, impaired neuronal plasticity, and neurotransmission in the prefrontal cortex and hippocampus (Felger and Lotrich 2013). Corroborating this observation, higher concentrations of proinflammatory cytokines, such as IL-6 and TNF-alpha, are observed more often in patients with depression than in healthy subjects (Dowlati et al., 2010).

**FIGURE 1:**
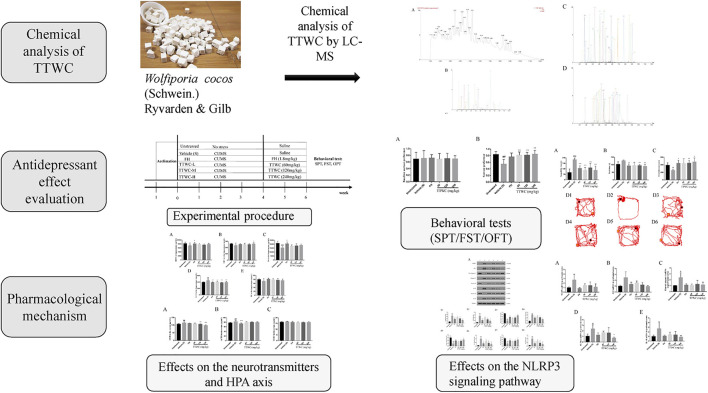
A schematic diagram of the research.

TTWC, as a natural anti-inflammatory substance (Jeong et al., 2014; Lee et al., 2017), may have the potential to be developed into new antidepressant drugs. In our previous study, we have revealed that TTWC can regulate levels of 5-hydroxytryptamine (5-HT), dopamine (DA), and other neurotransmitters in the hippocampus of sleep-deprived rats in the hippocampus, inhibit the hyperfunction of the hypothalamic-pituitary-adrenal (HPA) axis, and relieve anxiety symptoms. The present study is the first to describe the antidepressant-like effects of TTWC in a rat CUMS model. CUMS is a well-established procedure that induces depression-like behavior in rodents (Willner 2017). Accordingly, the sucrose preference test (SPT), forced swimming test (FST), and open field test (OFT) were performed in CUMS-induced rats to analyze antidepressant-like effects of TTWC after administration. The possible impact of TTWC on depression was studied from the perspective of regulation of neurotransmitter levels, the HPA axis, and neuroinflammation. The technical strategy used in this study is shown in Figure 1.

## Materials and Reagents

The triterpene compounds including polyporenic acid C (CAS: 465-18-9), 3-O-acetyl-16alpha-hydroxydehydrotrametenolic acid (CAS: 168293-14-9), poricoic acid B (CAS: 137551-39-4), dehydroeburicoic acid (CAS: 6879-05-6), poricoic acid A (CAS: 137551-38-3), 16alpha-hydroxydehydrotrametenolic acid (CAS: 176390-66-2), dehydrotumulosic acid (CAS: 6754-16-1), 16alpha-hydroxytrametenolic acid (CAS: 176390-68-4), pachymic acid (CAS: 29070-92-6), poricoic acid AM (CAS: 151200-92-9), 3-O-acetyl-16alpha-hydroxytrametenolic acid (CAS: 168293-13-8), dehydropachymic acid (CAS: 77012-31-8) (obtained from Push Bio-technology Co., Ltd., Chengdu, China) and dehydrotrametenolic acid (CAS: 29220-16-4) (obtained from Shanghai yuanye Bio-Technology Co., Ltd., Shanghai, China), were used as standards for triterpenes characterization of the TTWC. Fluoxetine hydrochloride (FH) was purchased from Lilly Suzhou Pharmaceutical Co., Ltd. (Suzhou, China). Commercial enzyme-linked immunosorbent assay (ELISA) kits for brain-derived neurotrophic factor (BDNF), 5-hydroxytryptamine (5-HT), dopamine (DA), glutamate (GLU), norepinephrine (NE), corticotropin-releasing hormone (CRH), adrenocorticotropic hormone (ACTH), corticosterone (CORT), interleukin-1beta (IL-1beta), interleukin-18 (IL-18), and tumor necrosis factor-alpha (TNF-alpha) interleukin-6 (IL-6) were purchased from Shanghai Fusheng Industrial Co., Ltd. (Shanghai, China). The primary antibodies used were rabbit polyclonal anti-beta-Actin (1:10,000) (Tianderui, China), rabbit monoclonal anti-NLRP3 (1:500) (Cell Signaling Technology, United States), mouse monoclonal anti-ASC (1:500) (Santa Cruz, United States), rabbit monoclonal anti-pro-caspase-1 (1:1,000) (Cell Signaling Technology, United States), rabbit polyclonal anti-caspase-1 (1:500) (Affinity Biosciences, United States), rabbit monoclonal anti-pro-IL-1beta (1:1,000) (Cell Signaling Technology, United States), rabbit polyclonal anti-IL-1beta (1:500) (Affinity Biosciences, United States), rabbit polyclonal anti-pro-IL-18 (1:500) (Affinity Biosciences, United States), and rabbit polyclonal anti-IL-18 (1:500) (Bioss, China) antibodies. The secondary antibodies used were HRP-goat anti-rabbit antibody (1:10,000) and HRP-goat anti-mouse antibody (1:10,000) (Aspen, China).

### Plant Materials

The sclerotia of *Wolfiporia cocos* were collected from Shitouju Town, Yingshan County, Huanggang City, Hubei Province, China, and identified by Prof. Xiao-Chuan Ye from the Hubei University of Chinese Medicine. The voucher specimen (No. 1807-1) was deposited in the herbarium of the Hubei Key Laboratory of Resources and Chemistry of Chinese Medicine, Hubei University of Chinese Medicine, Wuhan, China.

### Preparation of Extracts

The total triterpenes of *Wolfiporia cocos* were prepared as follows: Pulverized *Wolfiporia cocos* (4,320 g) were mixed with 80% ethanol and refluxed for 2 h. The supernatant was removed, and the residue was added to 80% ethanol for the second reflux. Supernatants were combined and concentrated by vacuum drying to produce the crude extract. After drying, the crude extract was magnetically stirred by washing thrice with distilled water. The residue was collected, soaked, and extracted in anhydrous ethanol thrice using an ultrasonic process. The mid-polarity fraction extract was dried in vacuum and then ground into powder, and the triterpene content in TTWC (20.40 g) was measured to be 66.33%.

### Phytochemical Characterization of the Total Triterpenes of Wolfiporia Cocos

The ACQUITY Ultra Performance LC (UPLC™) system coupled with a G2 QTOF™ system (Waters Technologies, Manchester, United Kingdom) was used in the present study. Chromatographic separation was performed on a Waters ACQUIYT UPLC R HSS C18 column (2.1 mm × 100 mm, 1.8 μm, Waters Corp., MA, United States). The column was maintained at 40°C. The mobile phase was composed of solvent A (0.1% formic acid aqueous solution), B (acetonitrile), and C (methanol). The gradient program was optimized as follows: 0–4 min, 42%–12% A, 55%–85% B and 3% C (The flow rate: 0.4–0.2 ml/min); 4–8 min, 12%–0% A, 85%–97% B and 3% C (The flow rate:0.2 ml/min); 8–10 min, 97%–100% B and 3–0% C (The flow rate: 0.2–0.4 ml/min); 10–12 min, 100% B (The flow rate: 0.4 ml/min); The injection volume of the reference compounds and samples was 2 μL. UPLC-QTOF-MS/MS analysis was performed using an ESI source operating in both positive and negative modes. The MS operating conditions were as follows: capillary voltage, 2,500 V; cone voltage, 40 V; ion source temperature, 100°C; desolvation gas, 500 L/h; and desolvation temperature, 500°C. The MS data were acquired in MS^E^ mode with a mass range of 50 to 1,200 m/z. Leucine enkephalin (500 pg/ml) was used as the lock mass to ensure accuracy and reproducibility.

### Animals

All experimental procedures were approved by the Animal Ethics Committee of the Hubei University of TCM. All efforts were made to minimize suffering and the number of animals used to produce reliable data. Male Sprague Dawley rats (180–220 g) were supplied by Sanxia University. (Animal license No: SCXK (E) 2017-0012). The animals and protocols for this study were approved by the Ethics Committee of the Animal Experiment Center of Hubei University of Traditional Chinese Medicine (No: HUCMS202011001). Rats were housed under standard conditions to adapt to the environment for 7 days before study initiation and had free access to food and water. The standard conditions for temperature (23 ± 2°C) and light (12:12-h light/dark cycle, lights on 8:00–20:00) were considered.

### Chronic Unpredictable Mild Stress Procedure

The CUMS procedure was conducted as previously described, with minor modifications (Willner 1997). Rats in the unstressed group were housed together with free access to water and food. The water was removed for 24 h before the SPT. CUMS rats were housed singly and subjected to the following stressors for four continuous weeks: 45°cage tilt (12 h), water and food deprivation (24 h), cold swimming at 4°C (5 min), damp bedding (24 h), cage rotation (20 min), tail clip (2 min), and electrical stimulation (2 min). The stressors were randomly scheduled once daily during CUMS procedure and every stressor presented four times within 4 weeks. The same stressor could not repeat on two consecutive days to ensure the unpredictability and avoid the adaptation by rats.

### Drug Administration and Treatment

**FIGURE 2:**
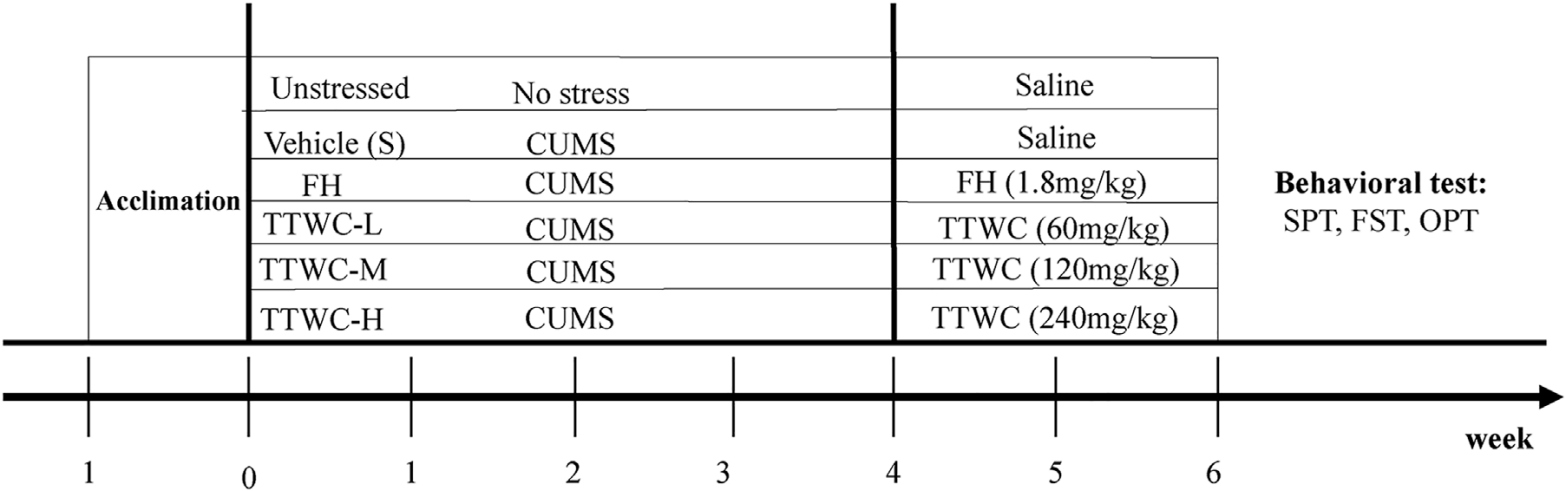
Schematic representation of the experimental procedure.

Before the CUMS procedure, the body weight and sucrose preference of each rat were tested. Based on results, rats were divided into two groups (48 rats in the CUMS-exposed group and 8 rats in the unstressed group). We ensured that there was no significant difference in body weight or sucrose preference among all groups. All rats in the CUMS-exposed group were subjected to CUMS for 4 weeks. After modeling, the body weight and open field total moving distance of each rat were measured. According to the results, the CUMS-exposed group was then divided into five groups: model, FH (1.8 mg/kg), TTWC-L (low), TTWC-M (middle), and TTWC-H (high) dose groups (60, 120, 240 mg/kg), with eight rats in each group. The dose of FH was converted from the clinical dose of 20 mg/d (Zhou et al., 2021). The unstressed and vehicle treated CUMS groups were administered saline every day, while the other groups received intragastric administration of the corresponding drugs for 2 weeks. The experimental procedure is shown in Figure 2.

## Behavioral Tests

### Depression-Like Behavior in Rats Was Determined Using Sucrose Preference Test, Forced Swimming Test, and Open Field Test

#### Sucrose Preference Test

The SPT was performed as previously described (Daodee et al., 2019). Rats were first habituated to sucrose solution (1% w/v) for 24 h: two bottles of 1% sugar water were placed in each cage, and one bottle was replaced with pure water 24 h later. After deprivation of food and water for 12 h, each rat was given free access to two bottles of solution (100 ml of tap water and 100 ml of 2% sucrose). The position of bottles was random. The test was completed 3 h later, and the sucrose preference was calculated using the following formula: sucrose preference (%) = sucrose consumption/(water consumption + sucrose consumption).

#### Forced Swimming Test

The FST was performed as previously described (Shi et al., 2019). Rats were placed in a cuboid with a water temperature of 24 ± 1°C. The immobility time of rats was monitored for 6 minutes. Each rat was forced to swim for 2 min, and the total duration of immobility was recorded in the remaining 4 min. Rats were considered to be immobile when they maintained floating in water without escaping from the cylinder.

#### Open Field Test

The OFT was carried out with reference to a previous study (Hassan et al., 2020). The experiment was conducted in four equal-sized square open boxes (40 × 40 × 50 cm). The test was carried out in a sound proof room under bright ambient light. A camera was installed directly above the central area of the four boxes. The rats were individually placed into center area, and the video analysis system was immediately activated to capture video automatically. The measuring time was 5 min for each rat, and the rats were placed in the center of the field and adapted to the field environment for 2 min. The rats were then recorded for 3 min. Floor surfaces and walls of the area were thoroughly cleaned with an alcohol solution and dried between each rat test. The single-subject tracking mode of the system was used to measure the rest time (rest time) and total moving distance (total distance).

### Tissue Preparation

After the last behavioral test, rats were sacrificed, and serum and brain tissue were collected. Blood was collected from the abdominal aorta, centrifuged (3,500 rpm) for 15 min at 4°C and the serum was stored at −80°C. The hippocampus and prefrontal cortex of 36 rats (six randomly selected from each group) were removed on ice and stored at −80°C after freezing with liquid nitrogen. The whole brains of 12 rats (two rats randomly selected from each group) were stored in 4% paraformaldehyde solution at 4°C for paraffin embedding.

### Hematoxylin and Eosin Staining

The whole brain was fixed with a 4% paraformaldehyde for more than 24 h, and the trimmed tissue was placed in the dehydration box. The dehydration box was placed in the dehydrator to dehydrate with a gradient of alcohol. Specimens were embedded in paraffin and sliced. Paraffin sections were baked, dewaxed, and hydrated. Sections were placed in distilled water, and a hematoxylin aqueous solution was added for 3 min. This step was followed by hydrochloride ethanol differentiation for 15 s, slightly washed with water, and placed in bluing buffer for 15 s. The specimen was then washed with running water and eosin-stained five 5 min, washed with running water, cleared, mounted, and the morphology and number of pyramidal cells and Nissl’s bodies in the hippocampus and prefrontal cortex were observed under a 400× microscope.

### Quantification of Brain-Derived Neurotrophic Factor, 5-HT, Dopamine, Glutamate, NE in *Hippocampus*, and Corticotropin-Releasing Hormone, Adrenocorticotropic Hormone, Corticosterone, Interleukin-1 Beta, Interleukin-18, Tumor Necrosis Factor-Alpha, Interleukin-6 in Serum by Enzyme-Linked Immunosorbent Assay

Rat hippocampus were used to determine the levels of BDNF and other neurotransmitters associated with CUMS-induced depression-like behavior. Serum was used to evaluate the activity of the HPA axis and proinflammatory cytokines related to CUMS-induced depression-like behavior. Levels of BDNF, 5-HT, DA, GLU, and NE in the hippocampus and levels of CRH, ACTH, CORT, IL-1beta, IL-18, TNF-alpha, and IL-6 in the serum of rats were measured using ELISA kits. The experiment was performed strictly under the ELISA instructions, and the concentration was normalized according to the standard curve.

### Detection of NLRP3, ASC, Pro-caspase-1, Caspase-1, Pro-Interleukin-1beta, Interleukin-1beta, Pro-Interleukin-18, and Interleukin-18 Content in Prefrontal Cortex by Western Blotting

Total protein was prepared from the prefrontal cortex of behavioral test rats. Approximately 40 μg of protein for each sample was loaded on electrophoresis and transferred onto polyvinylidene difluoride membranes. After blocking with 5% skimmed milk powder at room temperature for 1 h, the membrane was incubated with primary antibody at 4°C overnight, rinsed three times with Tris-buffered saline/Tween 20 (TBST) for 5 min, and incubated with HRP-conjugated secondary antibody for 30 min at 37°C. The membrane was rinsed four times with TBST for 5 min and then analyzed using a chemiluminescence imaging system. Relative expression of protein was detected by the ratio of the grayscale of target protein band/grayscale of beta-actin band. The mean intensity values were obtained from three independent experiments.

### Detection of NLRP3, ASC, Caspase-1, Interleukin-1beta, and Interleukin-18 mRNA in Prefrontal Cortex by Quantitative Reverse Transcription-Polymerase Chain Reaction

Quantitative Reverse Transcription-Polymerase Chain Reaction was performed according to the SYBR Green PCR kit instructions. The expression levels of ASC, caspase-1, IL-1beta, and IL-18 mRNA in the prefrontal cortex were determined. The sequences of the primers used for real-time PCR are listed in Table 1. The amplification procedure was as follows: 95°C for 30 s (95°C for 15 s, 60°C for 30 s) × 40. The data were analyzed using Nanodrop 2000 software, and the expression levels of ASC, caspase-1, IL-1beta, and IL-18 mRNA were determined.

**TABLE 1:** Primer information for the RT-PCR experiment.

Gene name	Primer sequence (5’→3′)
NLRP3	Primer F: CGG​ACT​GAC​CCA​TCA​ATG​CT
	Primer R: GCA​GCT​GAC​CAA​CCA​GAG​TT
ASC	Primer F: GCA​CAG​CCA​GAA​CAG​AAC​ATT​T
	Primer R: TGC​CAT​ACA​GAG​CAT​CCA​GC
Caspase-1	Primer F: CTG​GAG​CTT​CAG​TCA​GGT​CC
	Primer R: CTT​GAG​GGA​ACC​ACT​CGG​TC
IL-1beta	Primer F: CAG​CTT​TCG​ACA​GTG​AGG​AGA
	Primer R: TGT​CGA​GAT​GCT​GCT​GTG​AG
IL-18	Primer F: TAT​CGA​CCG​AAC​AGC​CAA​CG
	Primer R: GAT​AGG​GTC​ACA​GCC​AGT​CC
GAPDH	Primer F: TAC​TGT​TGT​CCA​GCT​ACG​GC
	Primer R: CGT​CCA​AAT​CCA​TTG​ATG​CCC

### Statistical Analysis

The experimental data were expressed as the mean ± standard deviations (SD). The experimental data were statistically analyzed and plotted using SPSS (version 25.0; SPSS Inc., Chicago, IL, United States) and GraphPad Prism 7 software (GraphPad Software Inc., San Diego, CA, United States). One-way analysis of variance (ANOVA) was used for comparison between groups, dunnett method was used for homogeneity of variance, Games-Howell method was used for heterogeneity of variance, where *p* < 0.05 indicated a significant difference, and *p* < 0.01 indicated a very significant difference.

## Results

### Composition Analysis of Total Triterpenes Of *Wolfiporia Cocos*

**FIGURE 3:**
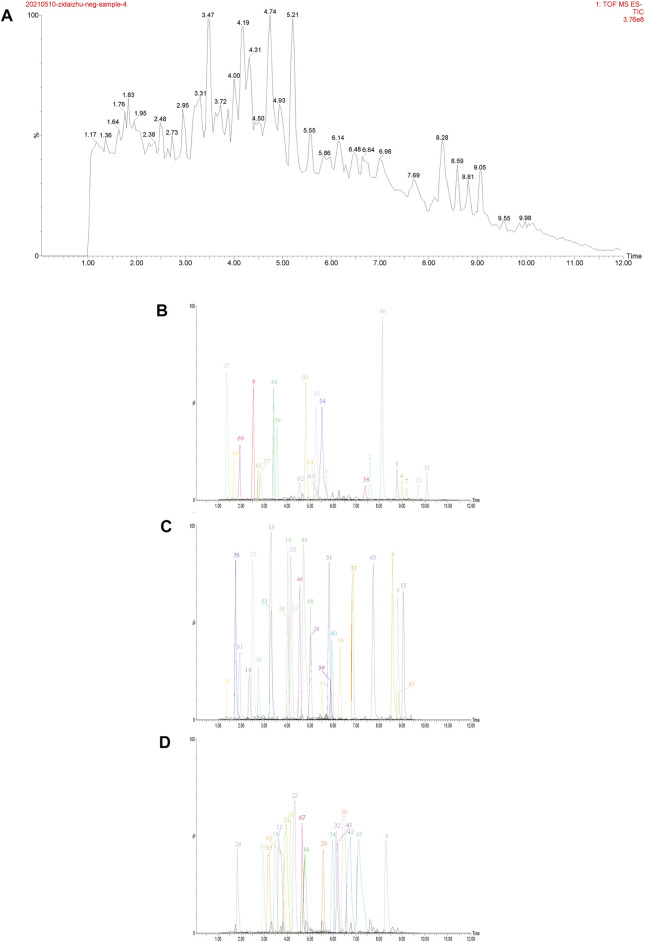
Total ion chromatogram (TIC) in negative ion mode obtained from UPLC-QTOF-MS/MS analysis of TTWC **(A)**, extracted ion chromatograms of 69 triterpenes detected by UPLC-QTOF-MS/MS in TTWC, intensity≤2e^6^ **(B)**, 2e^6^ < intensity≤8e^6^ **(C)**, 8e^6^ < intensity≤2e^7^ **(D)**.

Ultra-high performance liquid chromatography-quadropole time-of-flight mass spectrometry (UPLC-QTOF-MS/MS) determined components of TTWC in both positive and negative modes. Our results corroborated findings from previous literature and identified 69 chemical constituents in the negative ion mode (Feng et al., 2018; Zou et al., 2019). Extracted ion chromatograms of the 69 triterpenoids are shown in Figure 3.

### Effects of Total Triterpenes Of *Wolfiporia Cocos* on Depression-like Behavior Induced by Chronic Unpredictable Mild Stress in Rats

The depression-like behavior in CUMS rats was assessed to examine antidepressant-like effects of TTWC using SPT, FST, and OFT.

### Effects of Total Triterpenes of *Wolfiporia Cocos* on Sucrose Preference Test

**FIGURE 4:**
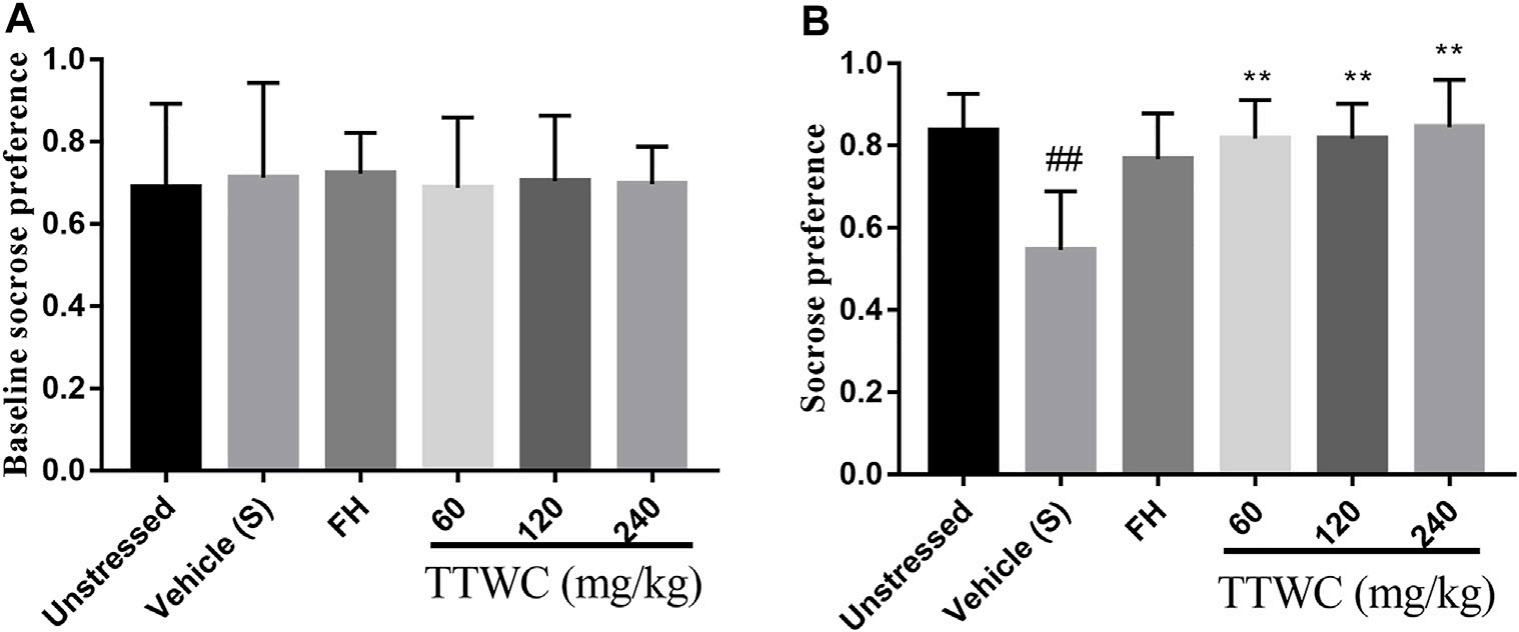
Effects of CUMS and TTWC treatment (60, 120, 240 mg/kg) on baseline sucrose preference **(A)** and SPT **(B)**. Values are expressed as (Mean ± SD, *n* = 6). ^**^ *p* < 0.01 vs. vehicle treated CUMS group. ^##^ *p* < 0.01 vs. unstressed group.

According to the results, sucrose preference remained consistent across all rats before the modeling process, as shown in Figure 4A. In the SPT (Figure 4B), sucrose preference among different groups significantly differed. Compared with that in the unstressed group (0.836 ± 0.091), CUMS rats showed significant decrease in the sucrose preference rate (0.574 ± 0.136, *p* < 0.01). After 14 days of administration, compared with that of the vehicle treated CUMS group, the sucrose preference rate of the other groups improved, and significant differences in the TTWC-L, TTWC-M, and TTWC-H groups (0.816 ± 0.094, *p* < 0.01; 0.817 ± 0.086, *p* < 0.01; 0.845 ± 0.116, *p* < 0.01) were observed.

### Effects of Total Triterpenes of *Wolfiporia Cocos* on the Immobility Time in Forced Swimming Test

**FIGURE 5:**
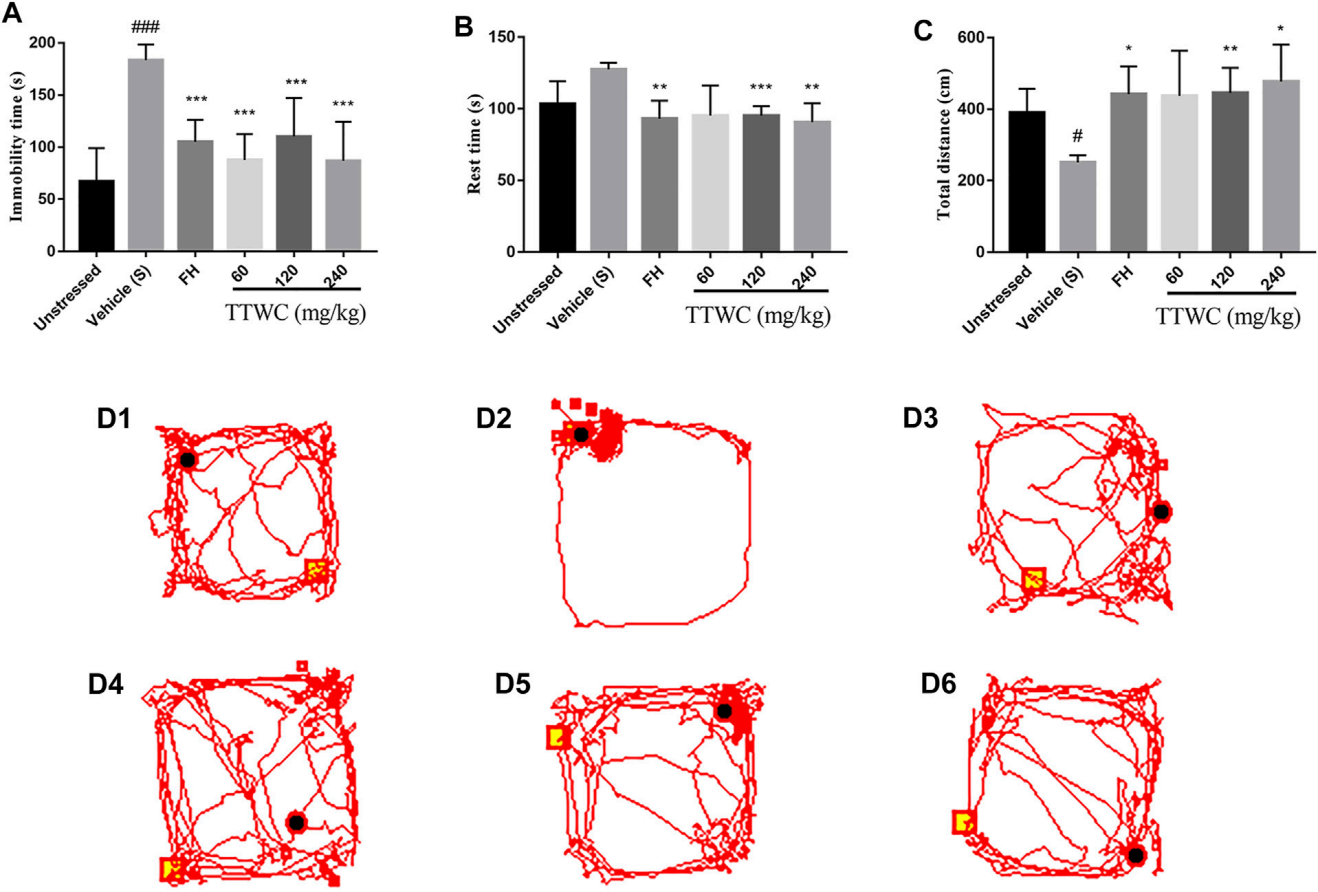
The Immobility time on FST **(A)**, the Rest time and Total distance of rats on OFT **(B,C)** and the map **(D1-D6)** of the movement trajectory of one rat in each group for 3 min in the open field. Values are expressed as (Mean ± SD, *n* = 6). ^*^ *p* < 0.05, ^**^ *p* < 0.01, ^***^ *p* < 0.001 vs. vehicle treated CUMS group. ^#^ *p* < 0.05, ^###^ *p* < 0.001 vs. unstressed group.

The effect of TTWC on FST in CUMS rats is shown in Figure 5A. The vehicle treated CUMS group’s immobility time (183.333 ± 15.029 (s)) during forced swimming was significantly higher than that in the unstressed group (66.833 ± 32.320 (s), *p* < 0.001), while FH and TTWC treatment shortened immobility time in FST compared to vehicle treated CUMS group. There were significant differences in the FH (105.000 ± 21.270 (s); *p* < 0.001), TTWC-L (87.667 ± 24.969 (s); *p* < 0.001), TTWC-M (110.167 ± 36.859 (s); *p* < 0.001), and TTWC-H groups (86.833 ± 37.584 (s); *p* < 0.001).

### Effects of Total Triterpenes of *Wolfiporia Cocos* on Open Field Test

Figures 5B,C highlight the effects of TTWC on the OFT in CUMS rats. Compared with the unstressed group (103.400 ± 15.808 (s); 390.233 ± 66.991 (s)), rest time increased (127.617 ± 4.391 (s)) and the total distance decreased significantly in the vehicle treated CUMS group (251.450 ± 19.767 (cm); *p* < 0.05). Compared with the vehicle treated CUMS group, the rest time in the FH (93.167 ± 12.552 (s); *p* < 0.01), TTWC-M (95.217 ± 6.563 (s); *p* < 0.001), and TTWC-H (90.700 ± 13.216 (s); *p* < 0.01) groups decreased significantly, while the total distance in the FH (442.117 ± 77.670 (cm); *p* < 0.05), TTWC-M (445.383 ± 70.391 (cm); *p* < 0.01), and TTWC-H (477.733 ± 102.677 (cm); *p* < 0.05) groups increased significantly. Figure 5D (1–6) demonstrates maps of the movement trajectory of one rat in each group for 3 min in the open field.

### Effects on Hippocampus and Prefrontal Cortex Tissue Morphology

**FIGURE 6:**
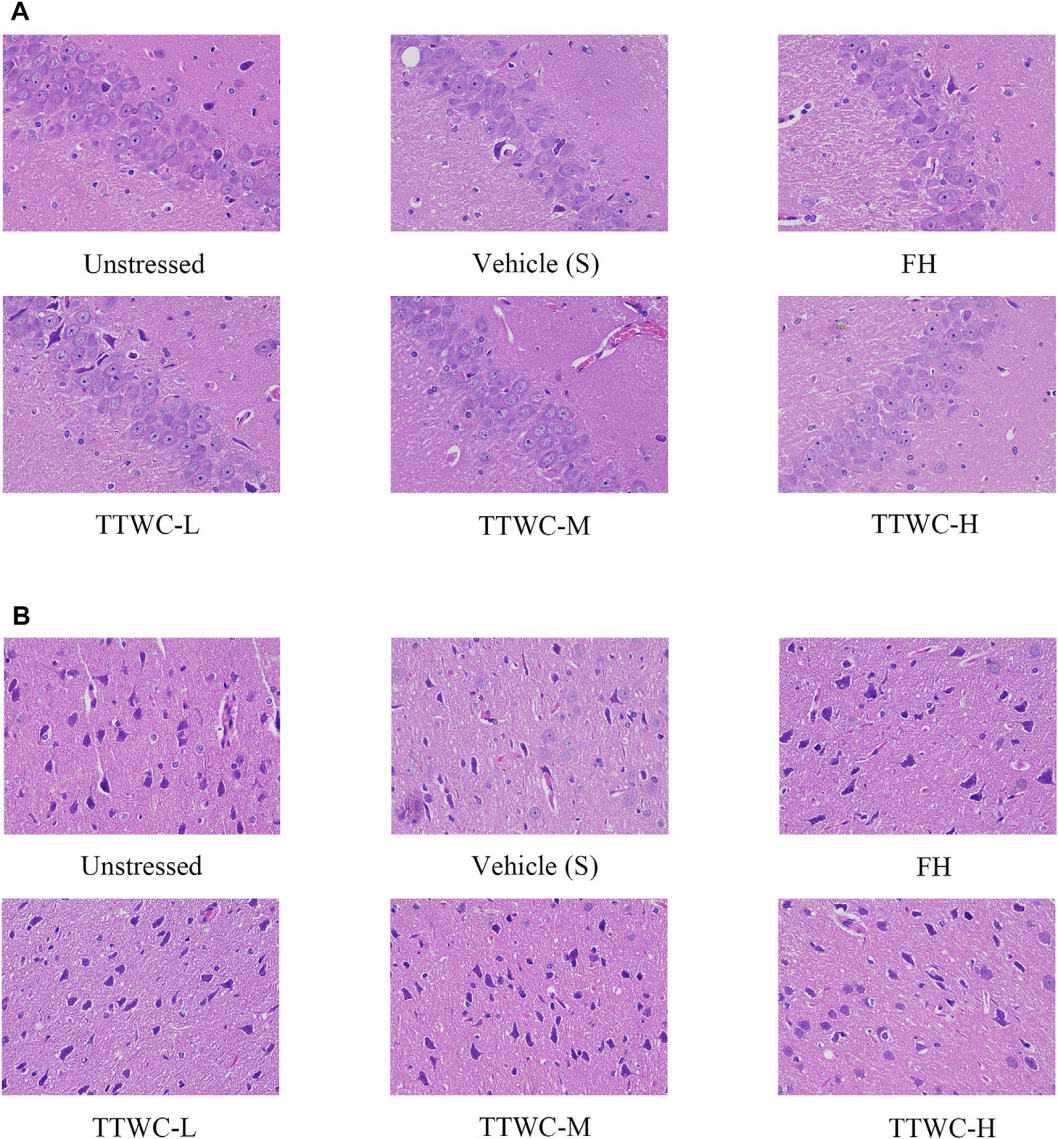
HE staining of the hippocampus CA3 area **(A)**, and the prefrontal cortex area **(B)** (400×).

HE staining of pyramidal cells in the CA3 region of the rat hippocampus, as viewed under a microscope, showed normal morphology and a tight arrangement of the pyramidal cells in the unstressed group. In contrast, the vehicle treated CUMS group demonstrated loosely arranged pyramidal cells with missing cells and a fuzzy structure. Moreover, the number of Nissl bodies in the prefrontal cortex and hippocampus was reduced in the vehicle treated CUMS group. In TTWC treated groups, the pyramidal cell morphology was improved, and the number of pyramidal cells and Nissl’s bodies were also increased to a certain extent. Figure 6 shows the effects on hippocampal and prefrontal cortex tissue morphology.

### Effects on the Brain-Derived Neurotrophic Factor and the Levels of Neurotransmitters (5-Hydroxytryptamine, Dopamine, Glutamate, Norepinephrine)

**FIGURE 7:**
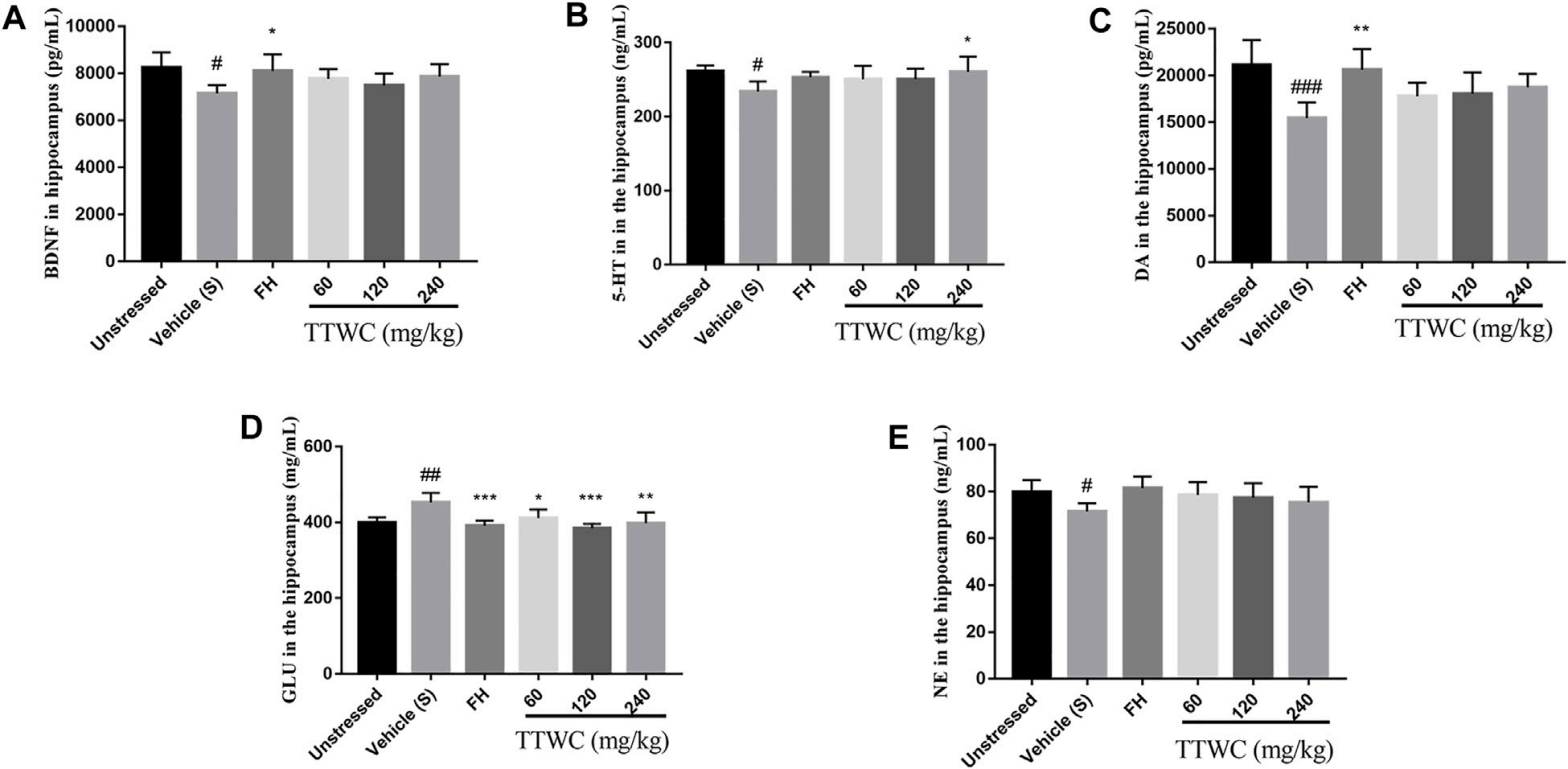
Effects of TTWC treatment on BDNF, 5-HT, DA, GLU and NE levels in hippocampus **(A, B, C, D, E)**. The levels of BDNF, 5-HT, DA, GLU and NE in the hippocampus of rats were tested by ELISA. Values are expressed as (Mean ± SD, *n* = 5). ^*^ *p* < 0.05, ^**^ *p* < 0.01, ^***^ *p* < 0.001 vs. vehicle treated CUMS group. ^#^ *p* < 0.05, ^##^ *p* < 0.01, ^###^ *p* < 0.001 vs. unstressed group.

Levels of BDNF and neurotransmitters in the hippocampus were assessed to determine the effect of TTWC in the treatment of depression. Levels of BDNF, 5-HT, DA, and NE in the hippocampus of CUMS-induced rats (7142.555 ± 350.856 (pg/ml), *p* < 0.05; 233.786 ± 13.485 (ng/ml), *p* < 0.05; 15432.659 ± 1,688.760 (pg/ml), *p* < 0.001; 71.536 ± 3.507 (ng/ml), *p* < 0.05) were significantly decreased compared with those of the unstressed group (8234.753 ± 653.382 (pg/ml); 261.689 ± 7.397 (ng/ml); 21131.856 ± 2671.791 (pg/ml); 79.776 ± 5.072 (ng/ml)), and levels of GLU increased significantly (452.209 ± 25.782 (mg/ml), *p* < 0.01) compared with unstressed group (399.186 ± 13.857 (mg/ml)) (Figures 7A–E). Treatment with TTWC increased BDNF levels and regulated neurotransmitters induced by CUMS. Compared with those of the vehicle treated CUMS group, levels of BDNF in the FH group were significantly increased (8103.601 ± 702.813 (pg/ml), *p* < 0.05). Although the results were not significantly different, levels of BDNF increased in TTWC treatment groups (7770.027 ± 408.575 (pg/ml); 7489.722 ± 493.698 (pg/ml); 7863.707 ± 523.775 (pg/ml)). Levels of 5-HT in the TTWC-H group were significantly increased (260.435 ± 20.555 (ng/ml), *p* < 0.01). DA levels in each group increased by varying degrees, with significant differences in the FH group (20611.449 ± 2237.574 (pg/ml), *p* < 0.01). The levels of DA in TTWC-L, TTWC-M and TTWC-H groups were up-regulated, although the up-regulation was not significantly different (17787.544 ± 1,452.384 (pg/ml); 18050.195 ± 2301.075 (pg/ml); 18744.344 ± 1,441.749 (pg/ml)). Upon treatment with FH and TTWC, levels of GLU significantly decreased compared with those of the vehicle treated CUMS group, with significant differences in all of the TTWC dose groups (411.169 ± 22.482 (mg/ml), *p* < 0.05; 384.920 ± 10.936 (mg/ml), *p* < 0.001; 397.590 ± 28.730 (mg/ml), *p* < 0.01). Compared with those of the vehicle treated CUMS group, NE levels were not significantly increased in the FH, TTWC-L, TTWC-M and TTWC-H groups (81.491 ± 4.935 (ng/ml); 78.556 ± 5.453 (ng/ml); 77.356 ± 6.249 (ng/ml); 75.353 ± 6.673 (ng/ml)).

### Effect on Hypothalamic-Pituitary-Adrenal Axis Activity in Rats

**FIGURE 8:**
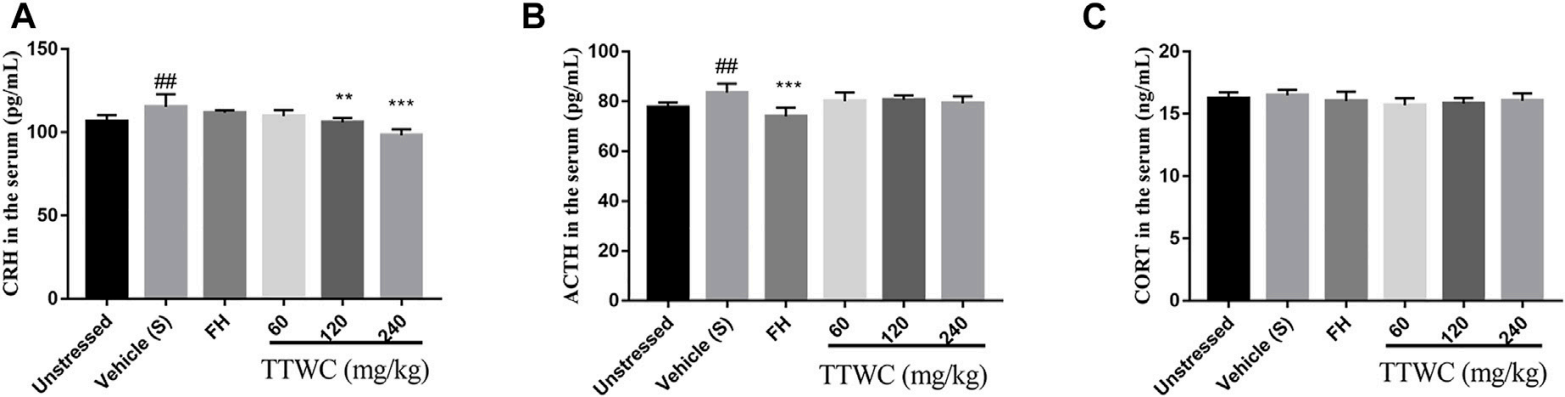
Effects of TTWC treatment on CRH **(A)**, ACTH **(B)** and CORT **(C)** levels (*n* = 6). The levels of CRH, ACTH and CORT in the serum of rats were tested by ELISA. Values are expressed as (Mean ± SD). ^**^ *p* < 0.01, ^***^ *p* < 0.001 vs. vehicle treated CUMS group. ^##^ *p* < 0.01 vs. unstressed group.

To investigate the regulatory effect of TTWC on the HPA axis activity in CUMS rats, levels of CRH, ACTH, and CORT in serum were determined by ELISA (Figures 8A–C). Serum levels of CRH and ACTH in the vehicle treated CUMS group (115.311 ± 7.621 (pg/ml), *p* < 0.01; 83.580 ± 3.559 (pg/ml), *p* < 0.01) were significantly higher than those in the unstressed group (106.583 ± 3.880 (pg/ml); 77.605 ± 2.002 (pg/ml)). Compared with those of the vehicle treated CUMS group, treatment with TTWC remarkably decreased CRH levels. There were significant differences in the TTWC-M (105.946 ± 2.784 (pg/ml), *p* < 0.01) and TTWC-H groups (98.142 ± 3.618 (pg/ml), *p* < 0.001). In addition, TTWC treatment reversed the CUMS-induced increase in serum ACTH levels. TTWC-L, TTWC-M and TTWC-H groups (80.084 ± 3.541 (pg/ml); 80.632 ± 1.753 (pg/ml); 79.249 ± 2.811 (pg/ml)) (*p* > 0.05) varied. Even CORT levels in serum in the CUMS group (16.496 ± 0.444 (ng/ml)) were not significantly higher than those in the unstressed group (16.241 ± 0.494 (ng/ml)), yet TTWC treatment groups reduced the serum level of CORT (15.684 ± 0.572 (ng/ml); 15.847 ± 0.428 (ng/ml); 16.050 ± 0.591 (ng/ml)) (*p* > 0.05).

### Effect of Total Triterpenes of *Wolfiporia Cocos* on the Level of Interleukin-1beta, Interleukin-18, Interleukin-6, and Tumor Necrosis Factor-Alpha in Serum

**FIGURE 9:**
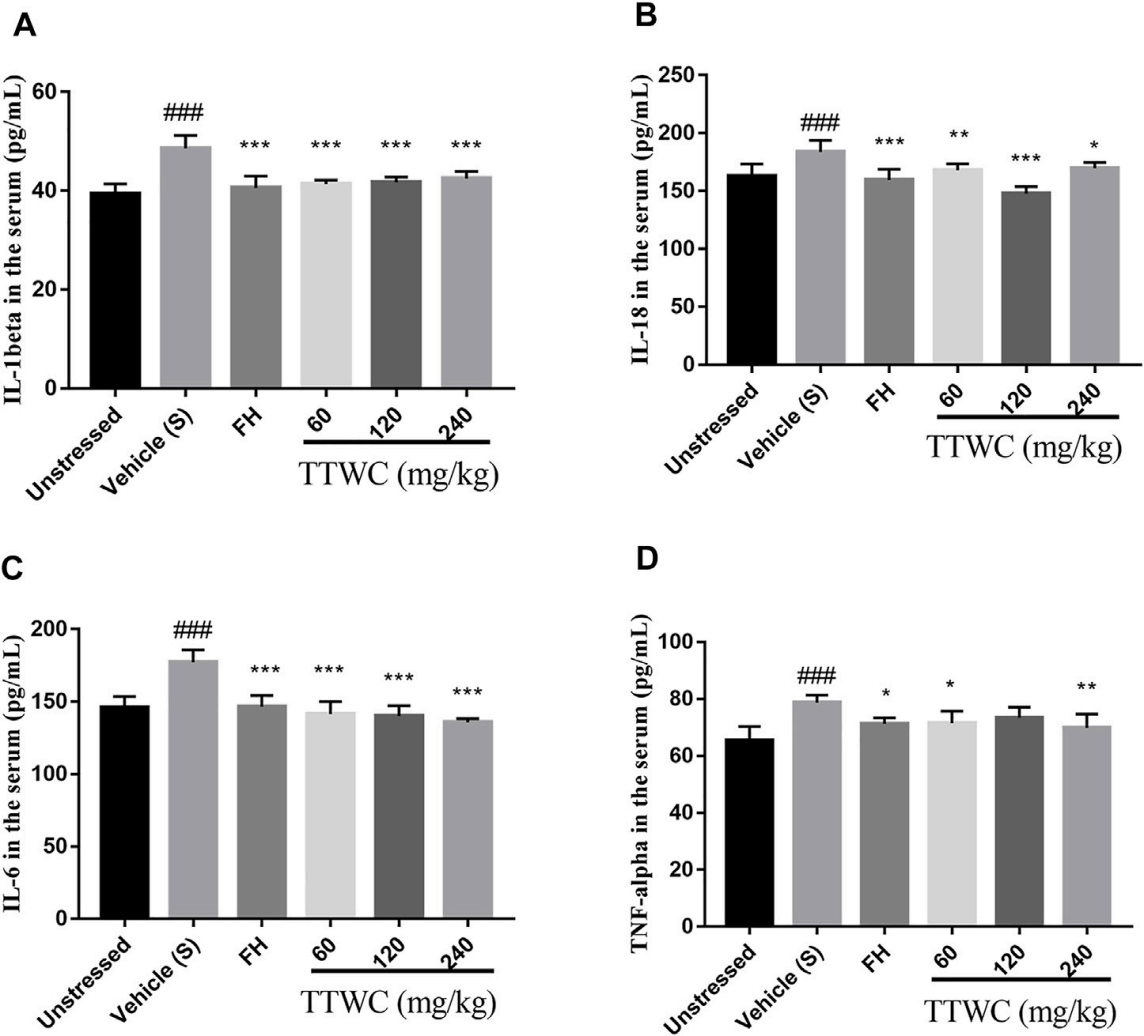
Effects of TTWC treatment on IL-1beta **(A)**, IL-18 **(B)**, IL-6 **(C)** and TNF-alpha **(D)** levels. The levels of IL-1beta, IL-18, IL-6 **(C)** and TNF-alpha **(D)** in the serum of rats were tested by ELISA. Values are expressed as (Mean ± SD, *n* = 6). ^*^ *p* < 0.05, ^**^ *p* < 0.01, ^***^ *p* < 0.001 vs. vehicle treated CUMS group. ^###^ *p* < 0.001 vs. unstressed group.

CUMS induced inflammation in different brain regions, with increased levels of cytokines including IL-1beta, IL-18, IL-6, and TNF-alpha in the serum (48.606 ± 2.601 (pg/ml), *p* < 0.001; 183.598 ± 10.324 (pg/ml), *p* < 0.001; 177.234 ± 8.463 (pg/ml), *p* < 0.001; 78.685 ± 2.567 (pg/ml), *p* < 0.001). According to the results of TTWC-L, TTWC-M, TTWC-H, treatment with different dose of TTWC reduced the levels of IL-1beta (41.353 ± 0.807 (pg/ml), *p* < 0.001; 41.742 ± 1.049 (pg/ml), *p* < 0.001; 42.454 ± 1.465 (pg/ml), *p* < 0.001), IL-18 (167.960 ± 5.619 (pg/ml), *p* < 0.01; 147.898 ± 6.032 (pg/ml), *p* < 0.001; 169.718 ± 4.953 (pg/ml), *p* < 0.05), IL-6 (141.507 ± 8.581 (pg/ml), *p* < 0.001; 140.173 ± 7.152 (pg/ml), *p* < 0.001; 135.921 ± 2.503 (pg/ml), *p* < 0.001), and TNF-alpha (71.506 ± 4.239 (pg/ml), *p* < 0.05; 73.495 ± 3.597 (pg/ml), *p* > 0.05; 69.885 ± 4.831 (pg/ml), *p* < 0.001) in the serum (Figures 9A–D).

### Effect of Total Triterpenes of *Wolfiporia Cocos* on the Level of NLRP3, ASC, Pro-caspase-1, Caspase-1, Pro-Interleukin-1beta, Interleukin-1beta, Pro-Interleukin-18 and Interleukin-18 Protein in the Prefrontal Cortex

**FIGURE 10:**
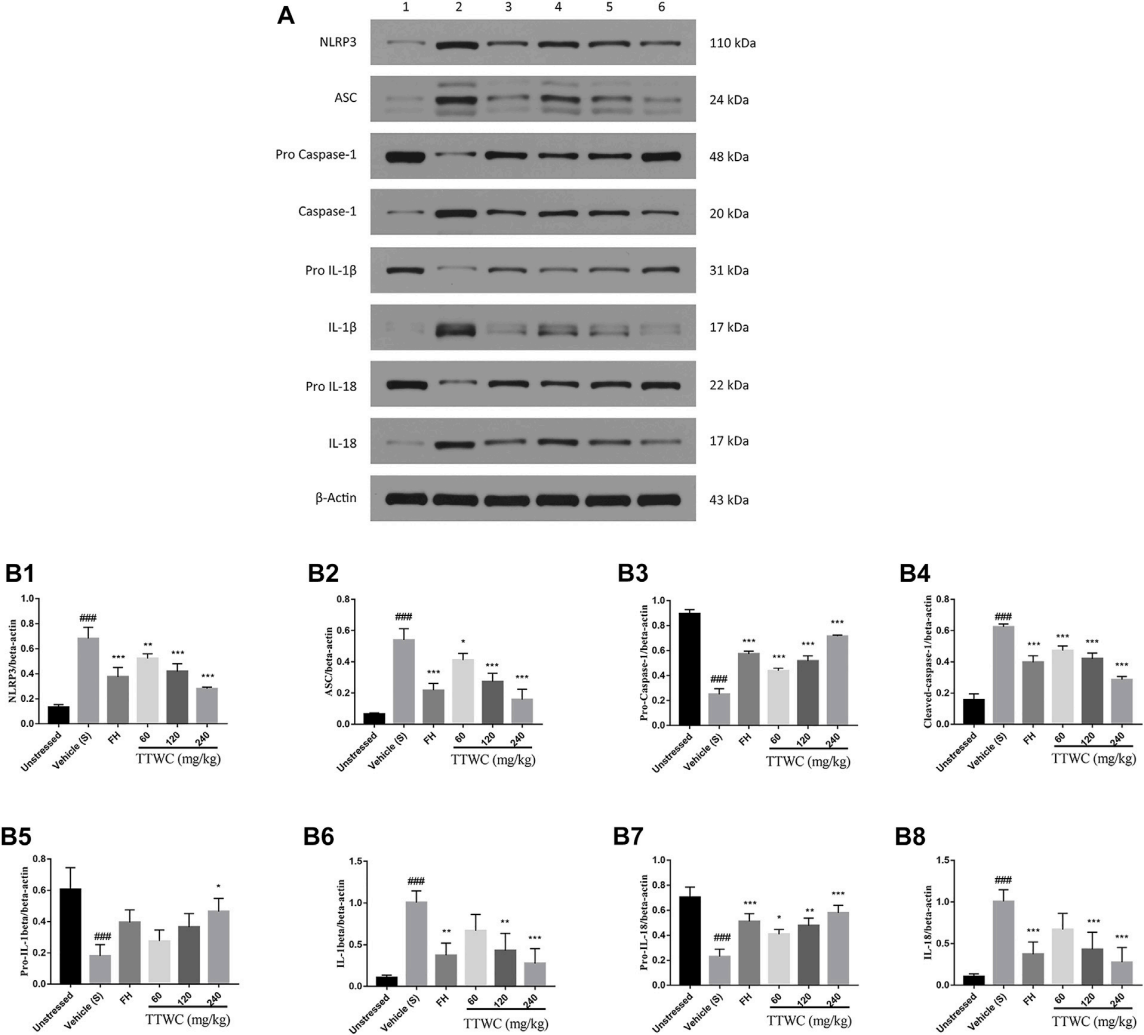
Effect of TTWC on the Level of NLRP3, ASC, pro-caspase-1, caspase-1, pro-IL-1beta, IL-1beta, pro-IL-18 and IL-18 protein in the prefrontal cortex **(B1-B8)** (Mean ± SD, *n* = 3). The results of western blot are shown in Figure 10A (1: unstressed group, 2: CUMS group, 3: FH group, 4: TTWC-L group, 5: TTWC-M group, 6: TTWC-H group). Values are expressed as (Mean ± SD). ^*^ *p* < 0.05, ^**^ *p* < 0.01, ^***^ *p* < 0.001 vs. vehicle treated CUMS group. ^###^ *p* < 0.001 vs. unstressed group.

Expression levels of NLRP3, ASC, pro-caspase-1, caspase-1, pro-IL-1beta, IL-1beta, pro-IL-18, and IL-18 in the prefrontal cortex of rats were semi-quantitatively analyzed using WB by comparing the ratio of the gray value of the beta-actin bands. Compared with unstressed group (0.132 ± 0.022; 0.065 ± 0.008; 0.157 ± 0.039; 0.105 ± 0.030; 0.087 ± 0.013), NLRP3, ASC, caspase-1, IL-1beta, and IL-18 protein in the prefrontal cortex were significantly increased (0.682 ± 0.089; 0.540 ± 0.072; 0.624 ± 0.019; 1.006 ± 0.140; 0.611 ± 0.074), and the pro-caspase-1, pro-IL-1beta, and pro-IL-18 levels were significantly decreased in the CUMS group (0.251 ± 0.044; 0.181 ± 0.072; 0.230 ± 0.059) compared with those of the unstressed group (0.895 ± 0.033; 0.607 ± 0.139; 0.704 ± 0.082) (*p* < 0.001) (Figure 10). The expression of NLRP3, ASC, pro-caspase-1, caspase-1, pro-IL-1beta, IL-1beta, pro-IL-18, and IL-18 was reversed in the FH (0.375 ± 0.075; 0.218 ± 0.044; 0.573 ± 0.023; 0.397 ± 0.042; 0.395 ± 0.079, *p* > 0.05; 0.373 ± 0.147; 0.511 ± 0.062; 0.343 ± 0.042) and TTWC-L (0.523 ± 0.037; 0.412 ± 0.042; 0.438 ± 0.021; 0.472 ± 0.030; 0.276 ± 0.071, *p* > 0.05; 0.670 ± 0.193, *p* > 0.05; 0.408 ± 0.038; 0.515 ± 0.037, *p* > 0.05), TTWC-M (0.419 ± 0.061; 0.274 ± 0.053; 0.517 ± 0.041; 0.420 ± 0.036; 0.366 ± 0.086, *p* > 0.05; 0.432 ± 0.205; 0.478 ± 0.059; 0.393 ± 0.034), and TTWC-H (0.281 ± 0.012; 0.158 ± 0.066; 0.715 ± 0.009; 0.286 ± 0.021; 0.465 ± 0.083; 0.276 ± 0.177; 0.579 ± 0.060; 0.240 ± 0.044) groups (*p* < 0.05, *p* < 0.01, or *p* < 0.001 except those were marked *p* > 0.05) compared with those of the CUMS group.

### Effect of Total Triterpenes of *Wolfiporia Cocos* on the Expression of NLRP3, ASC, Caspase-1, Interleukin-1beta, Interleukin-18 mRNA in the Prefrontal Cortex

**FIGURE 11:**
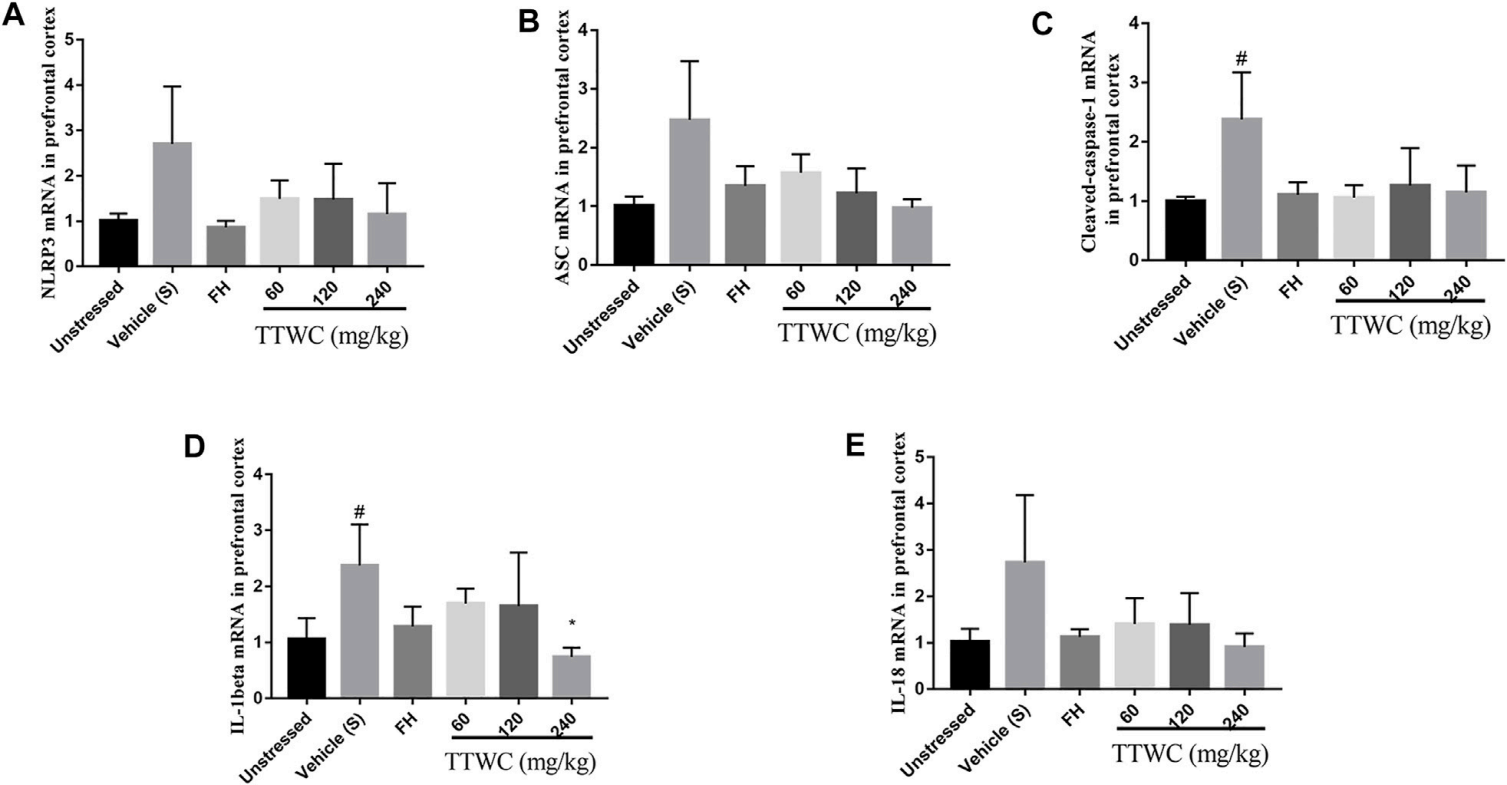
Effects of TTWC treatment on the mRNA levels of NLRP3, ASC, caspase-1, IL-1beta and IL-18 in the prefrontal cortex **(A,B,C,D,E)** (Mean ± SD, *n* = 6). ^*^ *p* < 0.05 vs vehicle treated CUMS group. ^#^ *p* < 0.05 vs unstressed group.

Similarly, RT-PCR analysis demonstrated that the expression of NLRP3, ASC, caspase-1, IL-1beta, and IL-18 mRNA in the prefrontal cortex of the vehicle treated CUMS group (2.705 ± 1.264, *p* > 0.05; 2.469 ± 0.999, *p* > 0.05; 2.377 ± 0.790, *p* < 0.05; 2.374 ± 0.733, *p* < 0.05; 2.731 ± 1.453, *p* > 0.05) was increased compared with those of the unstressed group (1.010 ± 0.156; 1.010 ± 0.155; 1.002 ± 0.071; 1.061 ± 0.373; 1.029 ± 0.274). In addition, the mRNA levels of caspase-1 and IL-1beta were significantly increased in the CUMS group (*p* < 0.05). In contrast, although most of the results were not significant, the expression of NLRP3 (1.493 ± 0.404; 1.476 ± 0.795; 1.156 ± 0.679), ASC (1.568 ± 0.318; 1.216 ± 0.431; 0.975 ± 0.139), caspase-1 (1.058 ± 0.210; 1.264 ± 0.629; 1.146 ± 0.451), IL-1beta (1.696 ± 0.265; 1.647 ± 0.958; 0.742 ± 0.168), and IL-18 (1.408 ± 0.555; 1.383 ± 0.689; 0.909 ± 0.295) mRNA in the TTWC treatment groups was reversed (Figures 11A–E).

## Discussion

CUMS, acknowledged as a putative animal model of depressive-like behavior and an imitation of life stress, is reliable and stable (Willner 1997; Mineur et al., 2006). Lack of pleasure is an essential manifestation of depression, and SPT is the gold standard for evaluating the degree of pleasure deficiency in depressed animals (Weiss 1997; Zhou et al., 2021). In this study, compared with rats in unstressed group, CUMS rats showed a lower degree of preference for sucrose after 4 weeks of CUMS treatment, which increased the sucrose preference index and demonstrated an antidepressant-like effect. The FST is commonly used to evaluate depression-like behavior in rodents, in which the immobility time reflects the degree of despair in rats (Porsolt et al., 1978). The immobility time in the vehicle treated CUMS group was significantly prolonged, which was reduced upon TTWC administration. The OFT was used to quantitatively evaluate rodents’ spontaneous activity exploration behavior and state of depression. Rats’ depressive-like behavior was assessed by their desire to explore the open field (Wang et al., 2014). We found that the desire to study the open field decreased in the vehicle treated CUMS group, the total moving distance decreased, and rest time was prolonged. However, these indices were reversed upon TTWC administration. Analysis of SPT, FST, and OFT results showed that rats in the vehicle treated CUMS group exhibited apparent depressive-like behavior, indicating the successful establishment of the model. Consistent with FH, an approved antidepressant, treatment with different doses of TTWC reversed these depressive-like behaviors and showed an evident antidepressant-like effect.

As shown in the previous studies, the hippocampus plays a vital role in the regulation of emotional learning and expression (Liu and Aghajanian 2008; Yao et al., 2020), while the prefrontal cortex is crucial in transforming emotional information into stress behavior and is involved in the neurological mechanisms of stress adaptation and pathology (Mcklveen et al., 2013), both of which are more easily affected in depression (Sheline et al., 1996; Luo et al., 2020). Among the findings of altered brain structure and function in depression, the most consistent finding is the reduced volume of the prefrontal cortex (PFC) and hippocampus (Savitz and Drevets 2009). In addition, studies have shown that the levels of neurotransmitter and inflammation in PFC and hippocampus are also consistent (Ashraf et al., 2019; Shen et al., 2019; Lama et al., 2021). Therefore, we observed the tissue morphology of PFC and hippocampus, and selected hippocampus and PFC tissues respectively to detect neurotransmitter levels and inflammation expression. It is generally recognized that depression is associated with an imbalance in the impairment of BDNF and certain neurotransmitters such as 5-HT, DA, GLU, and NE in the central nervous system (Tavakoli et al., 2018). BDNF is a potent regulator of long-term synaptic plasticity and mediates learning and memory. Low levels of BDNF signaling lead to hippocampal and cortical neural plasticity deficits, which are followed by neurodegenerative disorders, such as Alzheimer’s disease and major depression (Lu et al., 2013). The monoamine hypothesis is the first neurobiochemical theory of depression, which holds that deficiency of NE, 5-HT, or DA in the synaptic space occurs *in vivo*. It is also the leading cause of depression (Mcewen et al., 2010). Glutamate is the primary excitatory neurotransmitter in the brain. Once glutamate is released, glutamate receptors are stimulated excessively, leading to swelling and apoptosis of nerve cells and neurological disorders (Castillo et al., 1996). Therefore, we determined the levels of BDNF and neurotransmitters (5-HT, DA, GLU, and NE) in the hippocampus of rats. Our study revealed that in the CUMS group, the levels of BDNF, 5-HT, DA, and NE were decreased, and GLU levels were increased considerably. Their levels were reversed after intervention with FH and TTWC. Furthermore, the hippocampal and prefrontal cortex were improved morphologically and functionally by FH and TTWC administration. This observation indicated that TTWC might exert an antidepressant-like effect by regulating the levels of BDNF and neurotransmitters. In addition, we also hypothesized that it might promote the recovery of the hippocampus and prefrontal cortex.

The HPA axis, as a significant part of the neuroendocrine system, controls reactions to stress and regulates emotion (Swaab et al., 2005). Long-term chronic stress can lead to dysfunction of the HPA axis and increased secretion of CRH, ACTH, and CORT, which is considered to be closely involved in the pathogenesis of human depression (Fischer et al., 2017). The function of the HPA axis often returns to normal when depression patients or depressive animals receive effective treatment or when patients display spontaneous remission (Raone et al., 2007). Therefore, concentrations of CRH, ACTH, and CORT in the serum were measured. CRH and ACTH levels were increased in CUMS rats, with CORT levels being elevated to some extent. The administration of TTWC decreased CRH, ACTH, and CORT levels in the serum of CUMS rats. These results suggest that the inhibition of hyperfunction of the HPA axis may be an essential mechanism of the antidepressant-like effect of TTWC.

Growing evidence has revealed that suppressing the activation of NLRP3 and reducing the release of inflammatory cytokines plays a vital role in normalizing hyperactivity of the HPA axis (Lu et al., 2014). In addition, a positive correlation between neuroinflammation and depression has been reported in many clinical studies (Miller and Raison 2016). Stress from society, work, or life tend to cause elevated levels of proinflammatory cytokines such as IL-1beta, IL-18, IL-6, and TNF-alpha in both the periphery and brain, leading to sickness behavior syndrome; after treatment with antidepressants, the elevated levels of proinflammatory cytokines in some depression patients return to normal. (Miller et al., 2009). In the present study, CUMS rats showed increased levels of IL-1beta, IL-18, IL-6, and TNF-alpha in the serum. Notably, TTWC treatment decreased levels of IL-1beta, IL-18, IL-6, and TNF-alpha in the serum, indicating that the antidepressant-like effect of TTWC may be related to the inhibition of neuroinflammation in the prefrontal cortex. To further explore the mechanism of TTWC action, we investigated in depth the NLRP3 inflammasome, which controls the maturation of IL-1beta and IL-18.

The NLRP3 inflammasome is the most widely characterized NLR inflammasome complex. Canonical NLRP3 inflammasome complex is an intracellular protein complex consisting of the sensor NLRP3, the adaptor ASC, and pro-caspase-1. According to the previous literature, upon sensing danger signals, NLRP3 monomers, *via* their leucine-rich repeat motif domains, may interact with the pyrin domain of ASC, which then recruits the cysteine protease pro-caspase-1 *via* a caspase recruitment domain (Jo et al., 2016). Oligomerization of pro-caspase-1 proteins induces their proteolytic cleavage into active caspase-1 (Yang et al., 1998). Active caspase-1 cleaves precursor cytokines pro-IL-1beta and pro-IL-18, generating biologically active cytokines IL-1beta and IL-18 and, under certain conditions, to induce proptosis, a form of programmed inflammatory cell death (Guo et al., 2015). IL-1beta is a critical pro-inflammatory cytokine that can produce signal amplification cascades involving multiple inflammatory factors, thus aggravating inflammatory responses (LaRock et al., 2016). According to these findings, our study showed that the expression of NLRP3, ASC, Caspase-1, IL-1beta, and IL-18 was significantly reduced compared to those of the vehicle treated CUMS group. Levels of pro-caspase-1, pro-IL-1beta, and pro-IL-18 increased considerably in the FH and TTWC groups, suggesting that the inhibition of proinflammatory cytokine release induced by TTWC depends on the inhibition of the NLRP3 signaling pathway.

This study proved the antidepressant-like effect of TTWC for the first time, and the mechanism of its action was studied for the first time, which has laid the foundation for the development of new drugs for TTWC. We identified 69 components of TTWC by LC-MS analysis, the main constituents of which were the lanostane–type triterpene acids. We hypothesized that TTWC induces a comprehensive antidepressant-like effect via these components. Our research suggests that some components of TTWC may have more significant antidepressant-like effects. Generally, traditional Chinese medicines include complex components and act on numerous targets. Coupled with the interaction between the components, it is common that Chinese medicine does not show dose dependence (Li et al., 2020b; Ji et al., 2021; Ouyang et al., 2021). In this study, qRT-PCR and WB results show that the higher the dose of TTWC, the stronger the inhibitory effect of TTWC on the NLRP3 pathway. The reason is speculated to be because there are fewer components of TTWC entering brain regions, and one or more of these components may play a major role. Therefore, our subsequent studies will focus on these components entering brain regions. In contrast, the level of CRH, ACTH, CORT and neurotransmitters did not show a dose-dependence. In addition to the characteristics of traditional Chinese medicine, the reason for this phenomenon may be that TTWC does not directly regulate the HPA axis, but instead reduces neuroinflammation. According to a previous study, inflammation could stimulate HPA axis activity via both a direct action of cytokines on the brain and by inducing glucocorticoid resistance (Raison et al., 2006). Similarly, studies have shown that an anti-inflammatory agent can improve neurotransmitter levels by reducing neuroinflammation, possibly because it promoted synaptic plasticity in the hippocampus (Almeida et al., 2020; Lu et al., 2021).

In summary, here, we have analyzed the chemical composition of TTWC. We carried out an anti-depression experiment *in vivo* to identify how TTWC can improve depression-like behavior and alleviate neuronal damage in the hippocampus and prefrontal cortex by regulating the levels of neurotransmitters, the HPA axis, and the NLRP3 signaling pathway. Moreover, the NLRP3 pathway may be the main mechanism of action, mediating changes in neurotransmitters and HPA axis.

## Data Availability

The original contributions presented in the study are included in the article/Supplementary Files, further inquiries can be directed to the corresponding author.
