# Supplementary figures and images for: Total Triterpenes of Wolfiporia cocos (Schwein.) Ryvarden & Gilb Exerts Antidepressant-Like Effects in a Chronic Unpredictable Mild Stress Rat Model and Regulates the Levels of Neurotransmitters, HPA Axis and NLRP3 Pathway
Source: Front Pharmacol. 2022 Feb 14;13:793525. doi: 10.3389/fphar.2022.793525 (PMC8883346; doi:10.3389/fphar.2022.793525)

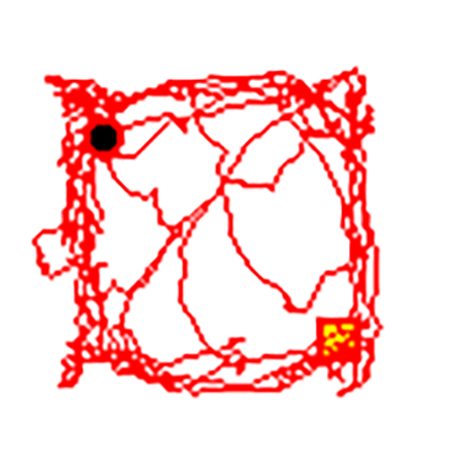

Supplement: Supplementary file 1 [file DataSheet1.ZIP › Supplementary Material/origin data/Behavioral/OFT/trajectory/Control.jpg]

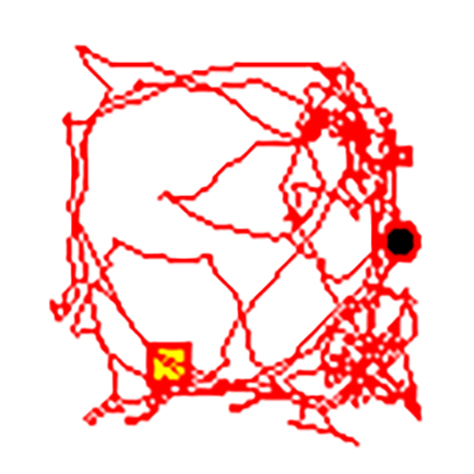

Supplement: Supplementary file 1 [file DataSheet1.ZIP › Supplementary Material/origin data/Behavioral/OFT/trajectory/FH.jpg]

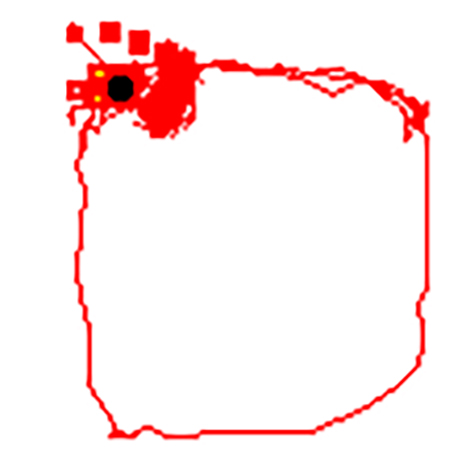

Supplement: Supplementary file 1 [file DataSheet1.ZIP › Supplementary Material/origin data/Behavioral/OFT/trajectory/Model.jpg]

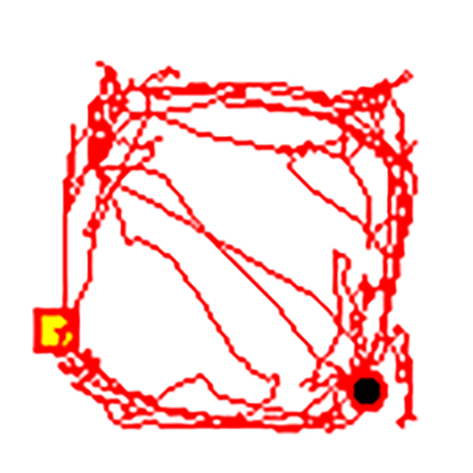

Supplement: Supplementary file 1 [file DataSheet1.ZIP › Supplementary Material/origin data/Behavioral/OFT/trajectory/TTPCH.jpg]

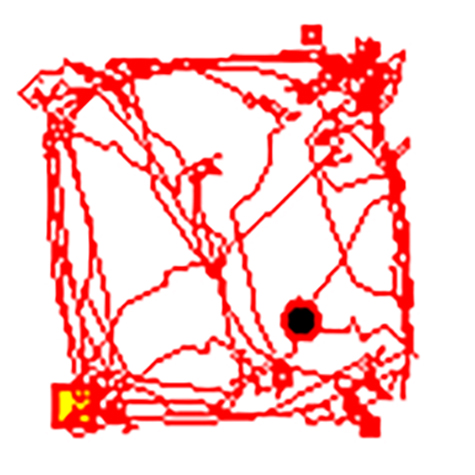

Supplement: Supplementary file 1 [file DataSheet1.ZIP › Supplementary Material/origin data/Behavioral/OFT/trajectory/TTPCL.jpg]

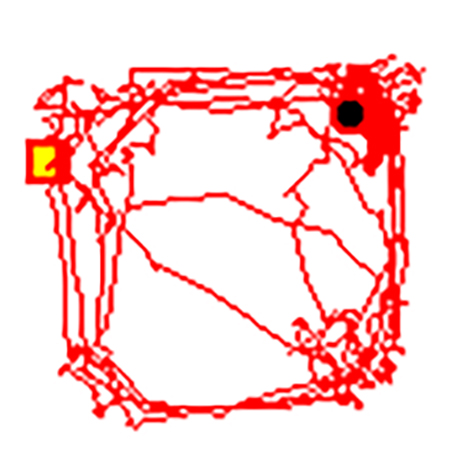

Supplement: Supplementary file 1 [file DataSheet1.ZIP › Supplementary Material/origin data/Behavioral/OFT/trajectory/TTPCM.jpg]

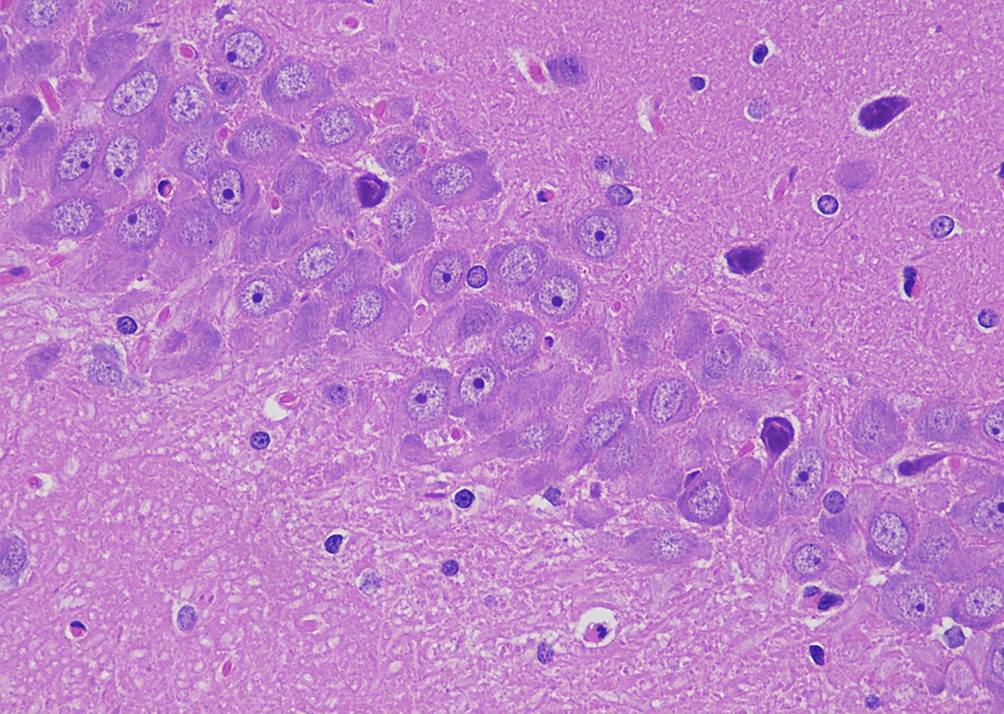

Supplement: Supplementary file 1 [file DataSheet1.ZIP › Supplementary Material/origin data/HE staining/Control hippocampus.jpg]

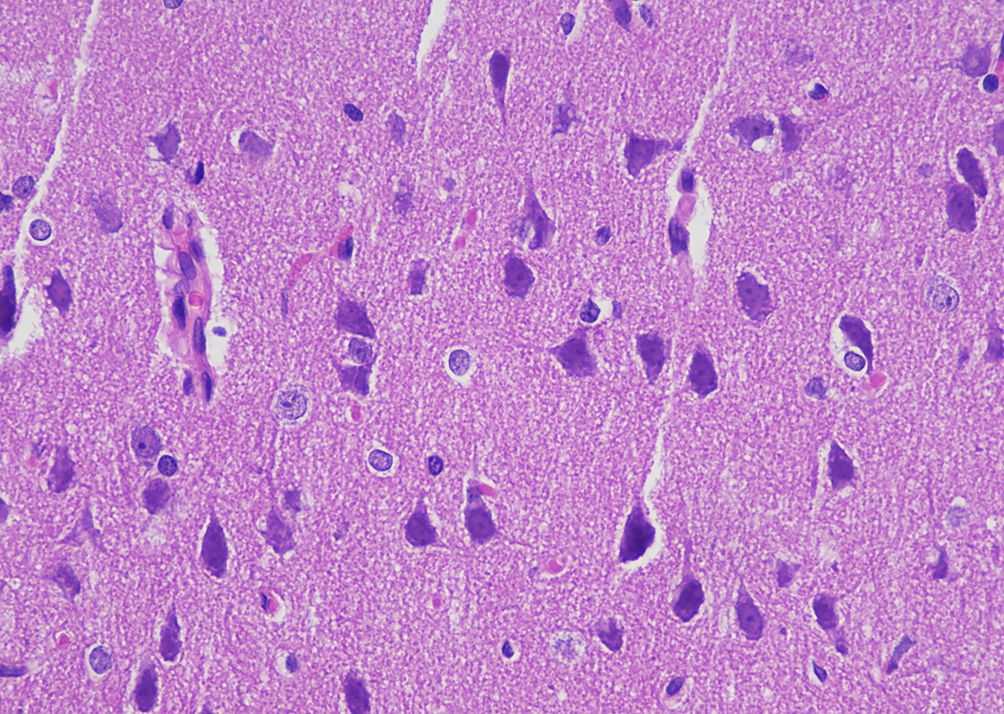

Supplement: Supplementary file 1 [file DataSheet1.ZIP › Supplementary Material/origin data/HE staining/Control prefrontal cortex.jpg]

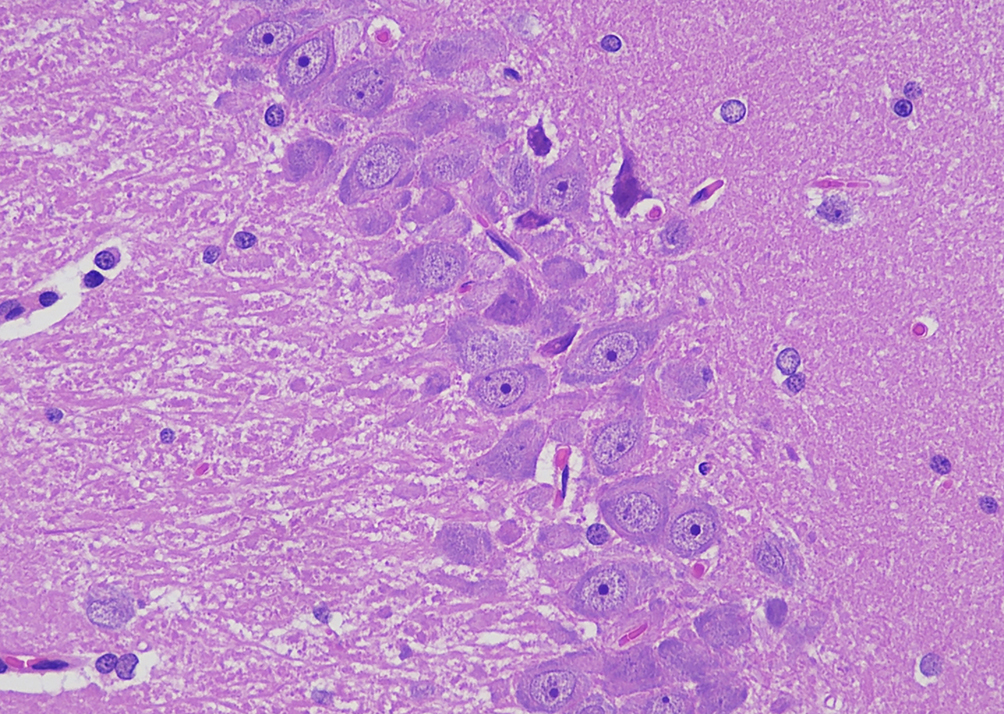

Supplement: Supplementary file 1 [file DataSheet1.ZIP › Supplementary Material/origin data/HE staining/FH hippocampus.jpg]

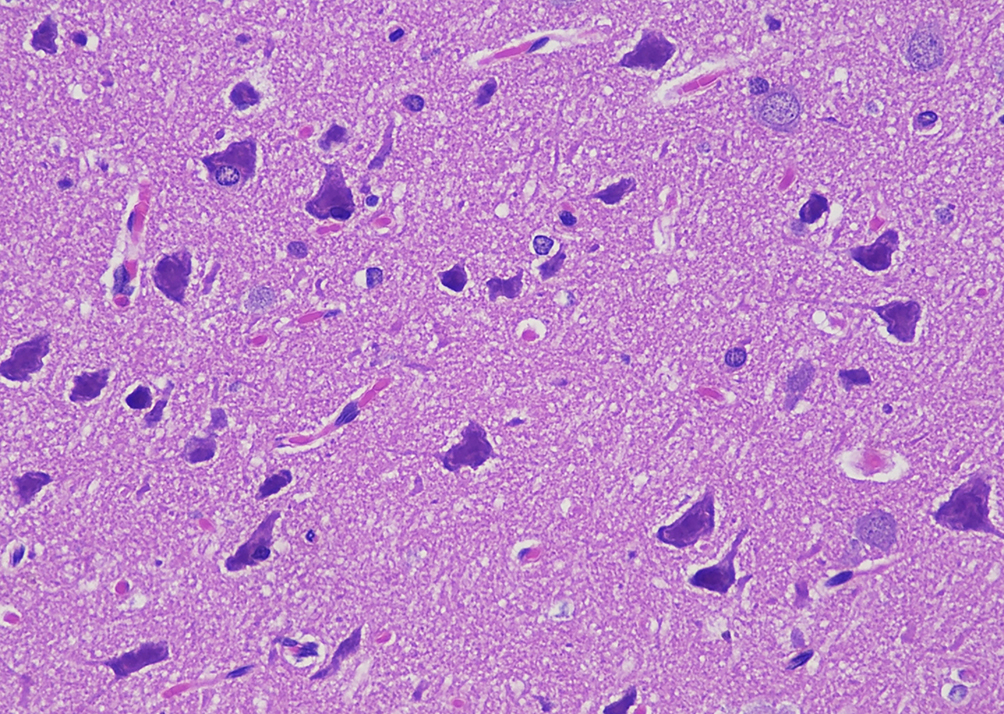

Supplement: Supplementary file 1 [file DataSheet1.ZIP › Supplementary Material/origin data/HE staining/FH prefrontal cortex.jpg]

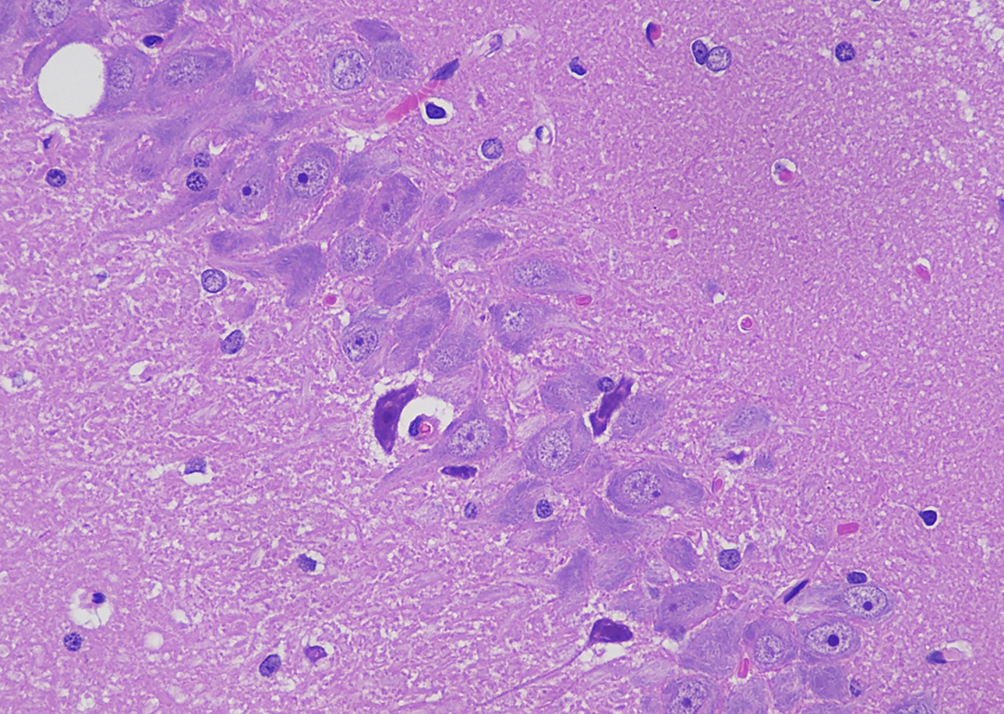

Supplement: Supplementary file 1 [file DataSheet1.ZIP › Supplementary Material/origin data/HE staining/Model hippocampus.jpg]

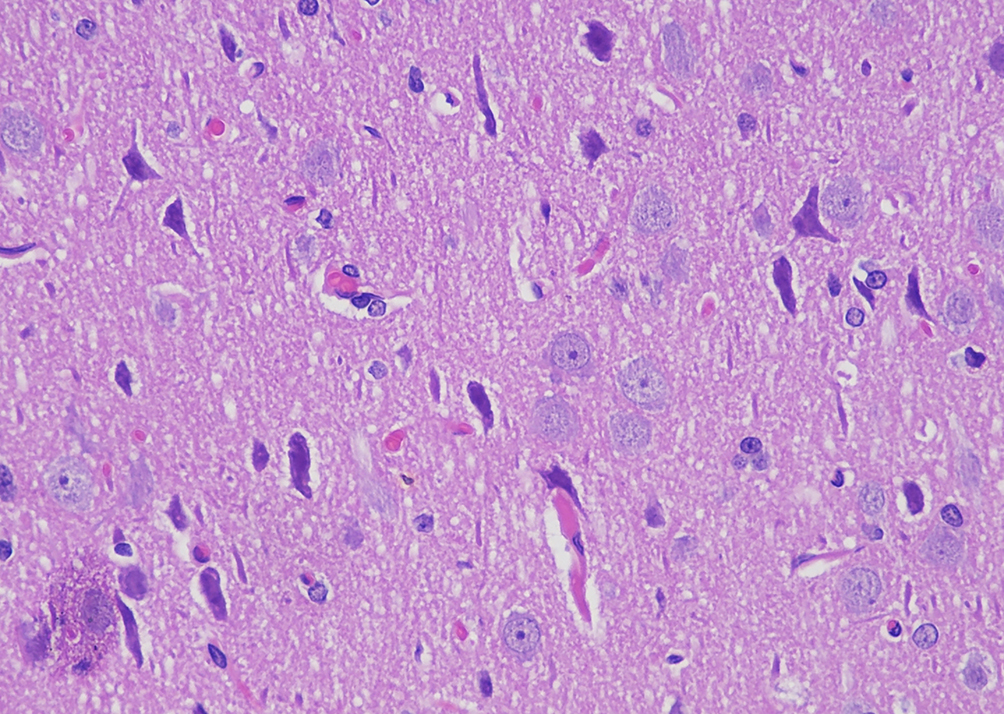

Supplement: Supplementary file 1 [file DataSheet1.ZIP › Supplementary Material/origin data/HE staining/Model prefrontal cortex.jpg]

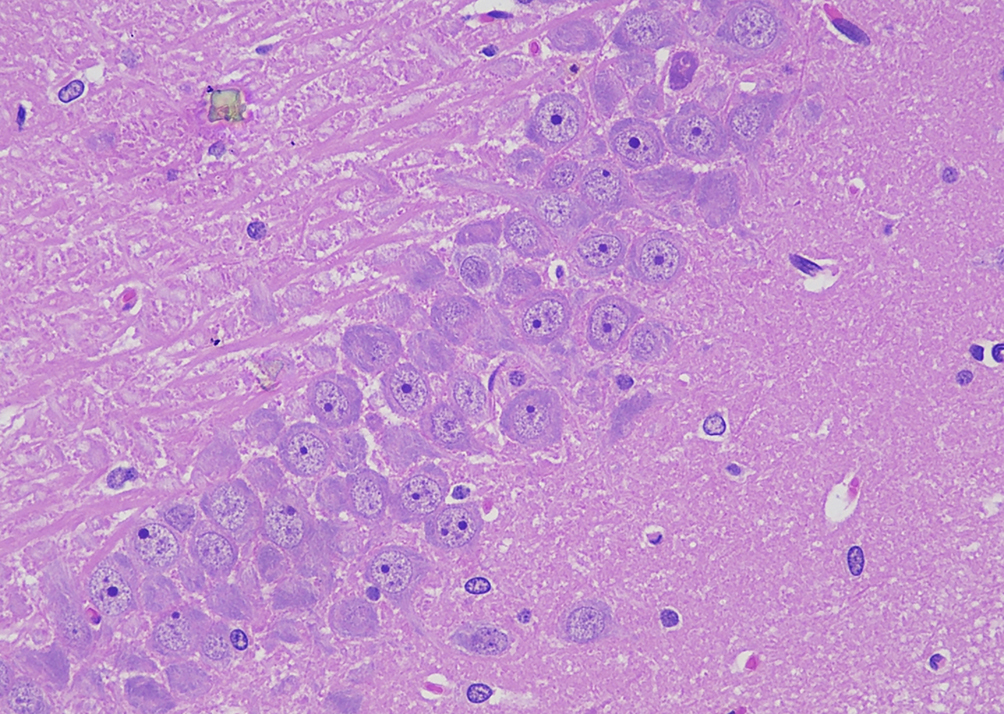

Supplement: Supplementary file 1 [file DataSheet1.ZIP › Supplementary Material/origin data/HE staining/TTPCH hippocampus.jpg]

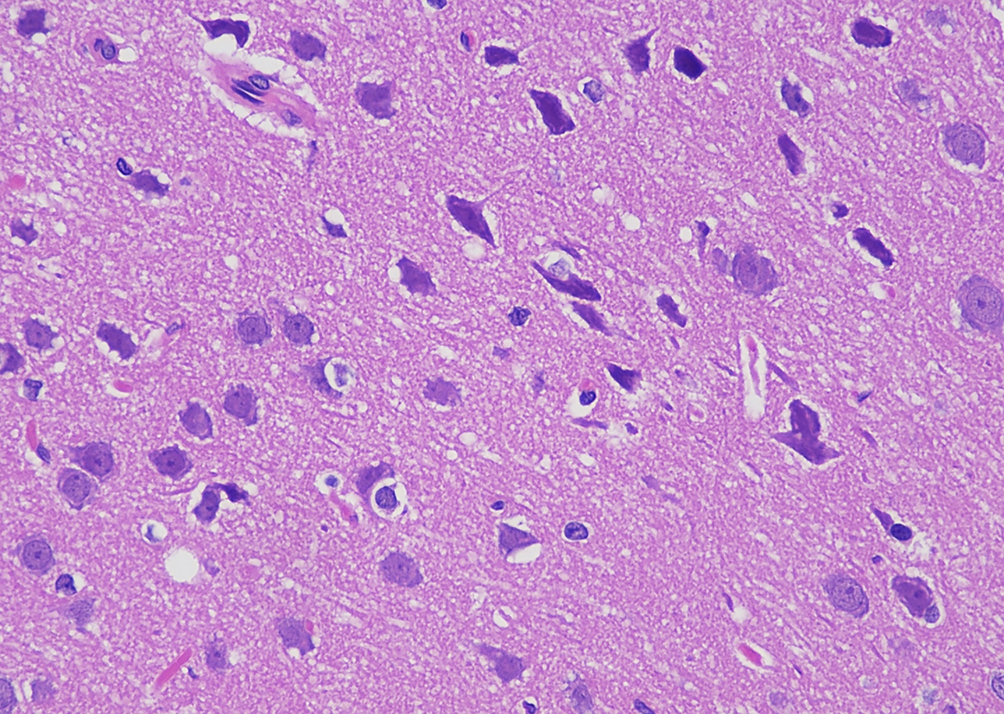

Supplement: Supplementary file 1 [file DataSheet1.ZIP › Supplementary Material/origin data/HE staining/TTPCH prefrontal cortex.jpg]

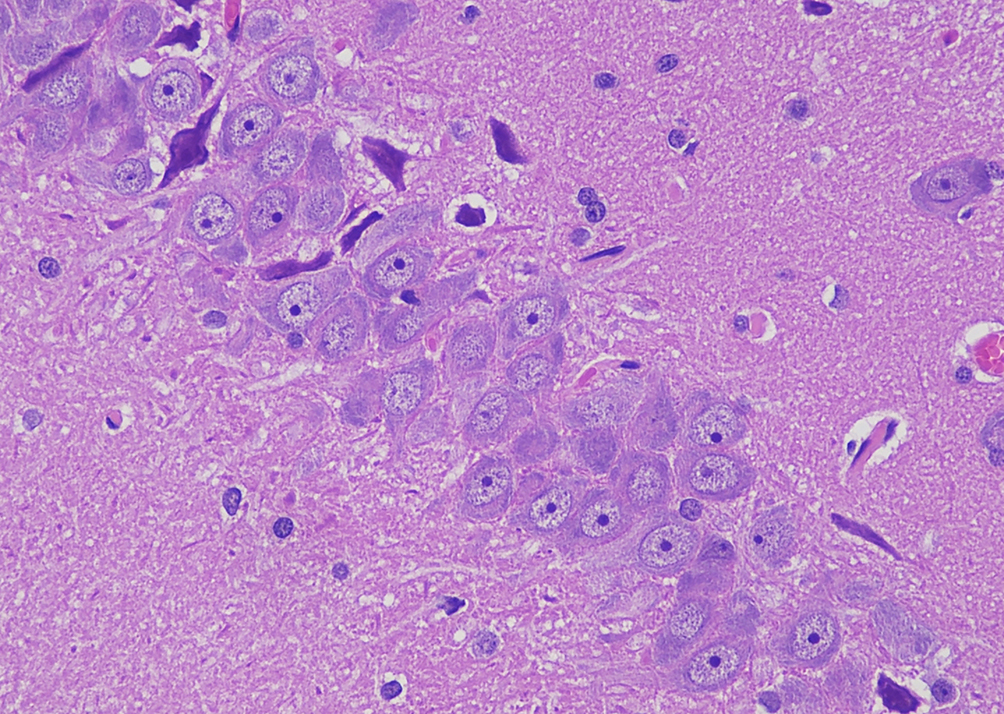

Supplement: Supplementary file 1 [file DataSheet1.ZIP › Supplementary Material/origin data/HE staining/TTPCL hippocampus.jpg]

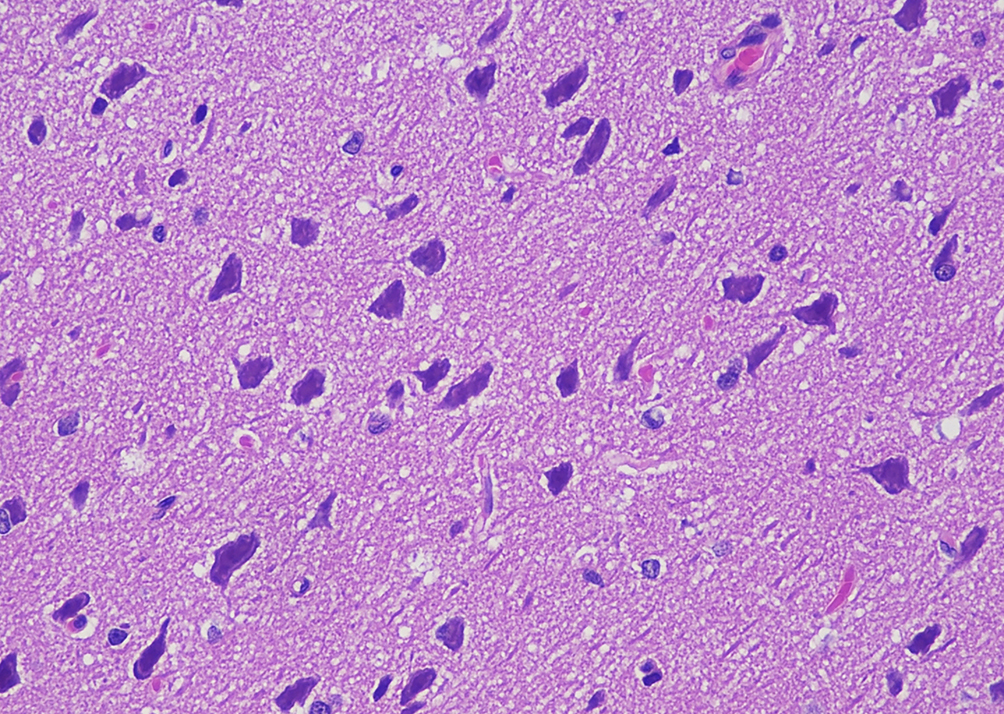

Supplement: Supplementary file 1 [file DataSheet1.ZIP › Supplementary Material/origin data/HE staining/TTPCL prefrontal cortex.jpg]

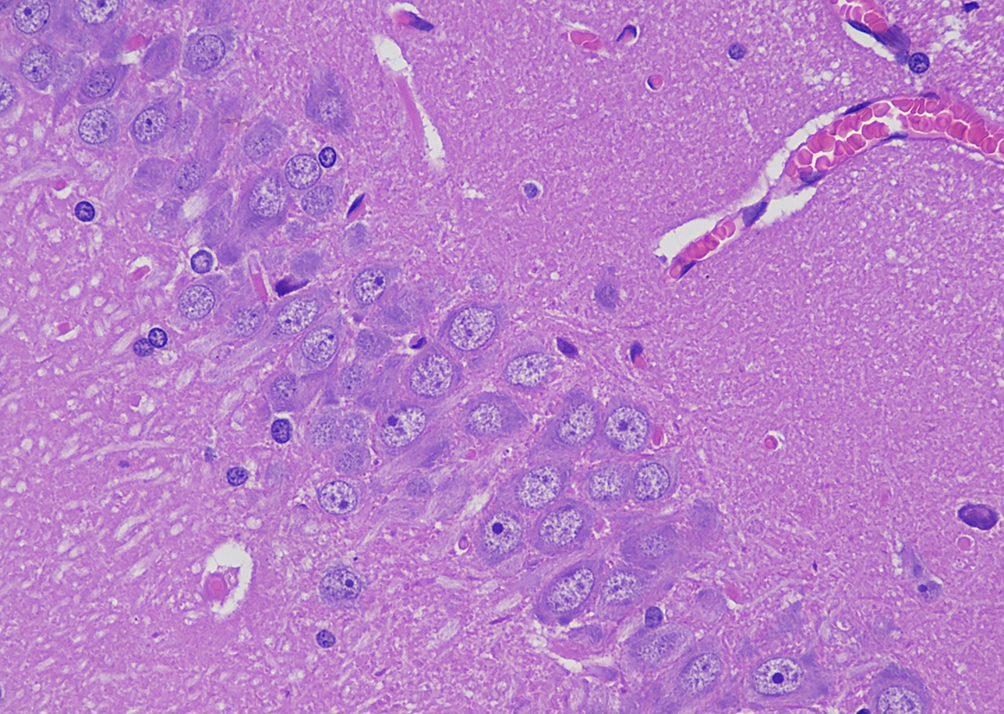

Supplement: Supplementary file 1 [file DataSheet1.ZIP › Supplementary Material/origin data/HE staining/TTPCM hippocampus.jpg]

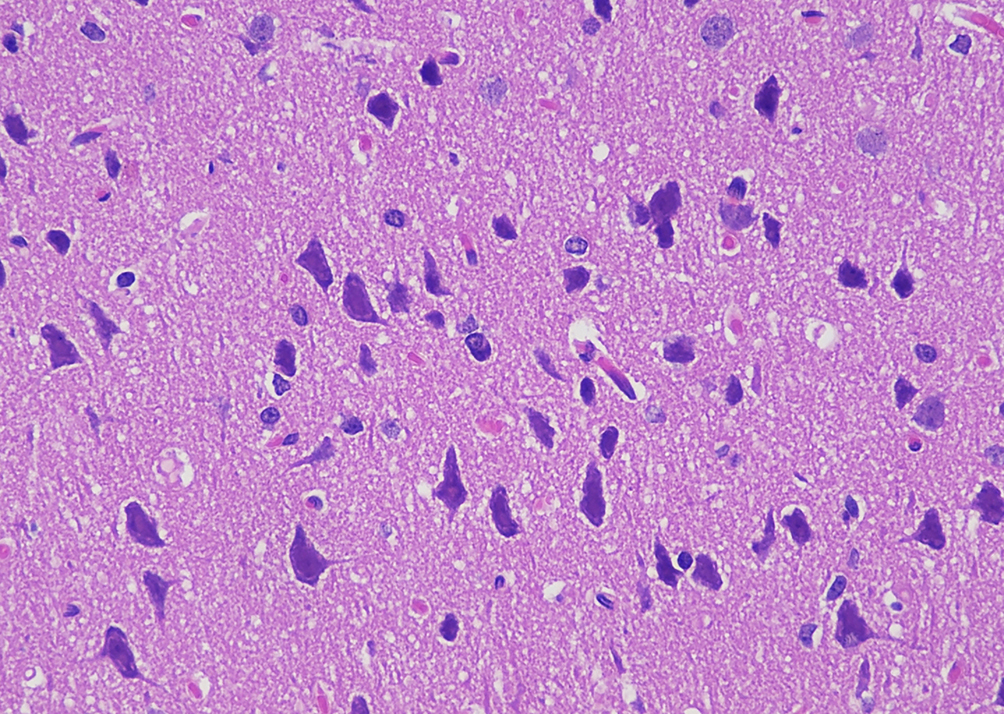

Supplement: Supplementary file 1 [file DataSheet1.ZIP › Supplementary Material/origin data/HE staining/TTPCM prefrontal cortex.jpg]

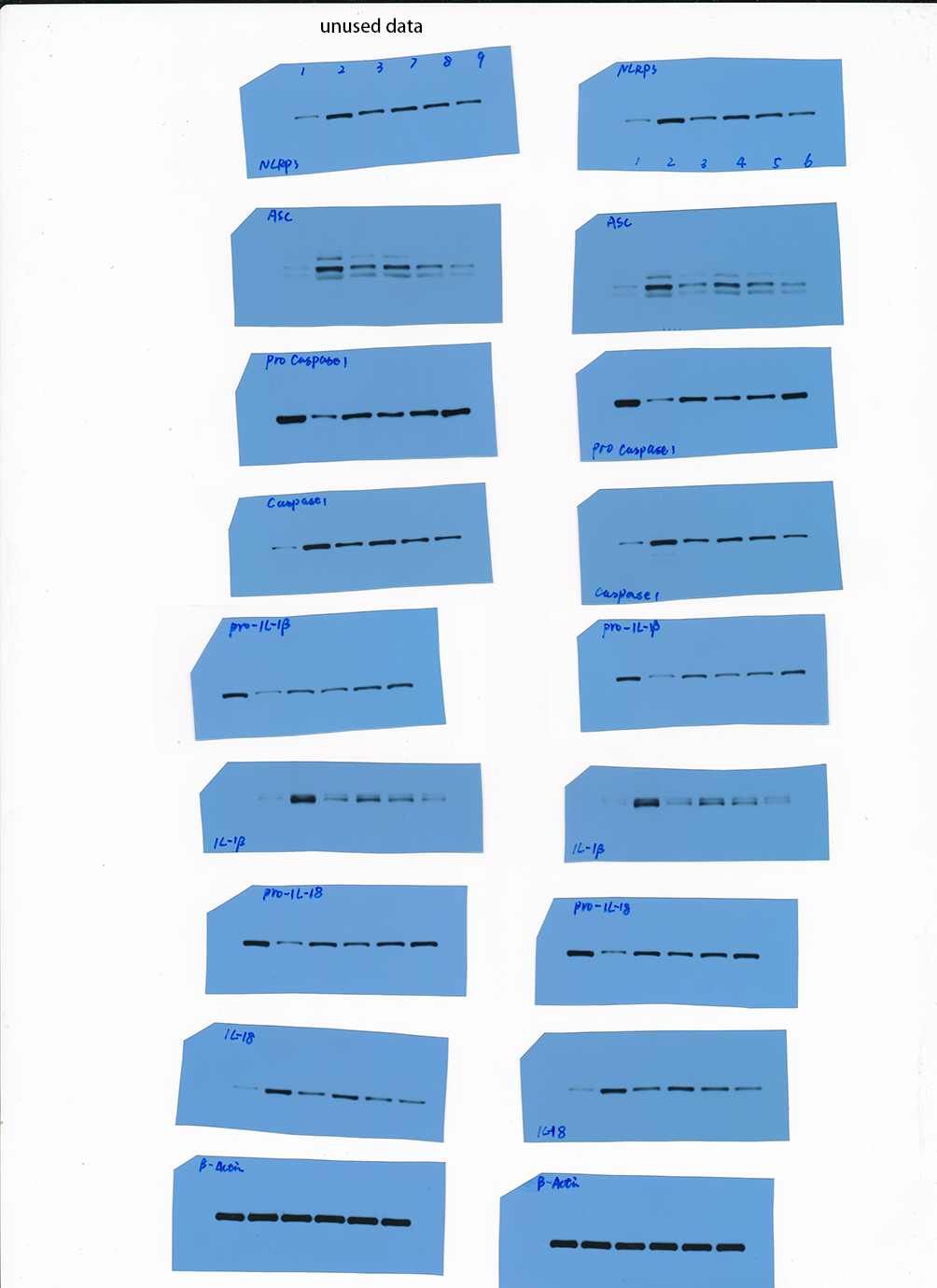

Supplement: Supplementary file 1 [file DataSheet1.ZIP › Supplementary Material/origin data/WB/WB1_20210222_0002.tif]

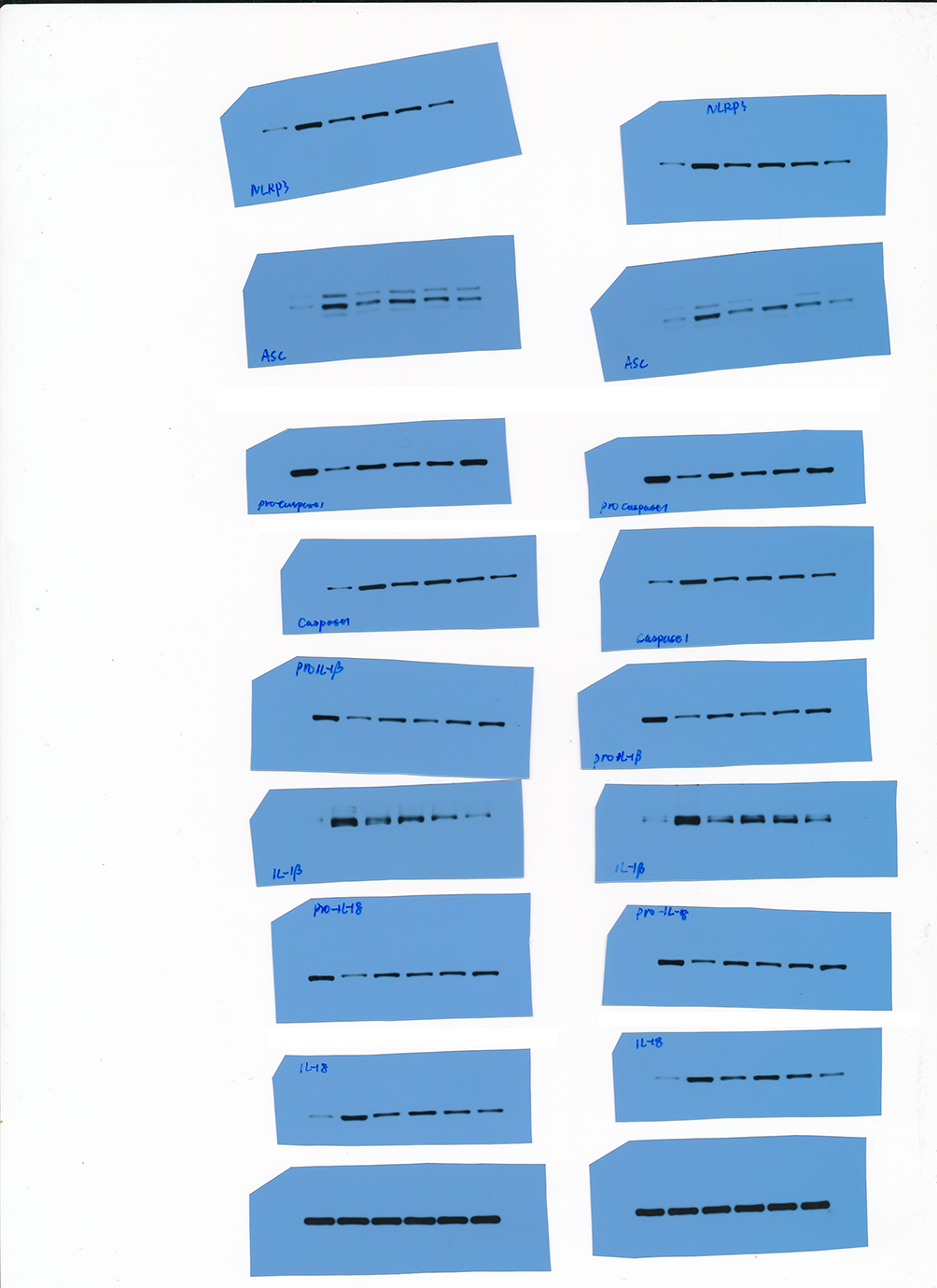

Supplement: Supplementary file 1 [file DataSheet1.ZIP › Supplementary Material/origin data/WB/WB1_20210302_0001.tif]
